# Establishing Criteria for Tumor Necrosis as Prognostic Indicator in Colorectal Cancer

**DOI:** 10.1097/PAS.0000000000002286

**Published:** 2024-07-15

**Authors:** Meeri Kastinen, Päivi Sirniö, Hanna Elomaa, Ville K. Äijälä, Henna Karjalainen, Vilja V. Tapiainen, Vesa-Matti Pohjanen, Janette Kemppainen, Katja Sliashynskaya, Maarit Ahtiainen, Jukka Rintala, Sanna Meriläinen, Tero Rautio, Juha Saarnio, Taneli T. Mattila, Outi Lindgren, Erkki-Ville Wirta, Olli Helminen, Toni T. Seppälä, Jan Böhm, Jukka-Pekka Mecklin, Anne Tuomisto, Markus J. Mäkinen, Juha P. Väyrynen

**Affiliations:** * Translational Medicine Research Unit, Medical Research Center Oulu, Oulu University Hospital, and University of Oulu, Oulu; † Department of Biological and Environmental Science, University of Jyväskylä, Jyväskylä; ‡ Department of Education and Research, Central Finland Health Care District, Jyväskylä; § Department of Pathology, Central Finland Health Care District, Jyväskylä; ∥ Department of Gastroenterology and Alimentary Tract Surgery, Tampere University Hospital, Tampere; ¶ Faculty of Medicine and Health Technology, Tampere University and Tays Cancer Centre, Tampere University Hospital, Tampere; # Department of Gastrointestinal Surgery, Helsinki University Central Hospital, University of Helsinki, Helsinki; ** Applied Tumor Genomics, Research Program Unit, University of Helsinki, Helsinki; †† Faculty of Sport and Health Sciences, University of Jyväskylä, Jyväskylä, Finland

**Keywords:** Tumor necrosis, prognosis, colorectal cancer, reproducibility, histopathology

## Abstract

Tumor necrosis has been reported to represent an independent prognostic factor in colorectal cancer, but its evaluation methods have not been described in sufficient detail to introduce tumor necrosis evaluation into clinical use. To study the potential of tumor necrosis as a prognostic indicator in colorectal cancer, criteria for 3 methods for its evaluation were defined: the average percentage method (tumor necrosis percentage of the whole tumor), the hotspot method (tumor necrosis percentage in a single hotspot), and the linear method (the diameter of the single largest necrotic focus). Cox regression models were used to calculate cancer-specific mortality hazard ratios (HRs) for tumor necrosis categories in 2 colorectal cancer cohorts with more than 1800 cases. For reproducibility assessment, 30 cases were evaluated by 9 investigators, and Spearman’s rank correlation coefficients and Cohen’s kappa coefficients were calculated. We found that all 3 methods predicted colorectal cancer-specific survival independent of other prognostic parameters, including disease stage, lymphovascular invasion, and tumor budding. The greatest multivariable HRs were observed for the average percentage method (cohort 1: HR for ≥ 40% vs. <3% 3.03, 95% CI, 1.93-4.78; cohort 2: HR for ≥ 40% vs. < 3% 2.97; 95% CI, 1.63-5.40). All 3 methods had high reproducibility, with the linear method showing the highest mean Spearman’s correlation coefficient (0.91) and Cohen’s kappa (0.70). In conclusion, detailed criteria for tumor necrosis evaluation were established. All 3 methods showed good reproducibility and predictive ability. The findings pave the way for the use of tumor necrosis as a prognostic factor in colorectal cancer.

Colorectal cancer (CRC) is one of the most common causes of cancer deaths worldwide and the incidence is continuously increasing.^1^ Predictive and prognostic factors for CRC include the tumor-node-metastasis (TNM) classification, mismatch repair (MMR) status, lymphatic or vascular invasion, perineural invasion, *BRAF* or *RAS* mutations, grade, and tumor budding.^2,3^ To refine the prognostic classification, additional parameters have been investigated, and tumor necrosis has proven to be an independent prognostic factor in several studies.^4–6^ These studies have mostly visually estimated tumor necrosis percentage and then categorized it based on certain cutoff values.^5^ However, the optimal evaluation method is not clear, and the specifics of the evaluation methods have not been described in sufficient detail to introduce tumor necrosis evaluation into clinical use.

Tumor budding is an example of a histopathologic parameter that has been successfully adapted into clinical decision-making, based on its strong prognostic value and reproducible criteria for its evaluation, established by the International Tumor Budding Consensus Conference (ITBCC).^7^ Tumor necrosis could also be evaluated from hematoxylin and eosin-stained samples and it does not require any complex staining or analysis methods. However, more detailed criteria are required to firmly establish it as a useful prognostic parameter. In this study, we aimed to address this by thoroughly describing 3 methods for tumor necrosis evaluation and comparing their prognostic value in more than 1800 CRC cases and reproducibility among 9 pathologists and researchers.

## MATERIALS AND METHODS

### Patients

Two cohorts were analyzed, cohort 1 from Central Finland Central Hospital (N=1343) and cohort 2 from Oulu University Hospital (N=1011). Cohort 1 has been retrospectively collected and includes patients from 2000 to 2015.^8^ Cohort 2 has been prospectively collected since 2006. It was previously described from 2006 to 2014^9,10^ and has now been extended until 2020. Both cohorts consist of CRC patients who have undergone tumor resection and from whom adequate tumor samples have been available. Patients who received preoperative radiotherapy or chemoradiotherapy were excluded from the analysis (cohort 1, N=243; cohort 2, N=235), and after their exclusion, there were 1100 patients for cohort 1 and 776 patients for cohort 2. Patients who died within 30 days after surgery were further excluded from survival analysis (cohort 1, N=37; cohort 2, N=5). Colorectal cancer-specific survival was used as the study endpoint, and it was defined as the time from operation to colorectal cancer-related death or the end of follow-up.

In survival analysis, the follow-up was limited to 10 years, considering that most colorectal cancer deaths occur within that time. The median follow-up time for censored cases was 10 years (IQR 7.3 to 10) for cohort 1 and 5.6 years (IQR 3.7 to 9.3) for cohort 2. The total number of deaths in cohort 1 was 531, of which 296 were cancer deaths. Cohort 2 had a total of 244 deaths, of which 135 were cancer deaths.

### Histopathologic Analyses

Tumor resection specimens were fixed using 10% formalin, embedded in paraffin, and hematoxylin and eosin (H&E) stained. Basic tumor parameters such as TNM stage, grade, and invasion status have been previously collected from tumor samples in both cohorts.^8,11,12^ TNM stage was determined by the Union for International Cancer Control/The American Joint Committee on Cancer (UICC/AJCC) criteria. The grade was assessed using the WHO criteria. MMR status and *BRAF* V600E mutation status were analyzed using immunohistochemistry.^8,11,12^ For cohort 2, neuroendocrine differentiation was determined using immunohistochemistry. Tissue microarrays were stained for chromogranin A (clone LK2H10, code MA5-13096, ER1, 1:400; Thermo Fisher, Waltham, MA) and synaptophysin (clone MRQ-40, code 336R-95, ER2, 1:20, Cell Marque, Rocklin, CA) with Leica Bond RX automated stainer and BOND Polymer Refine Detection kit (Leica DS9800). For antigen retrieval BOND Epitope Retrieval Solution 2 (Leica AR9640, 30min, 100°C) was used. The expression levels of chromogranin A (CHGA) and synaptophysin (SYP) were assessed as the percentage of positive tumor cells. These percentages were then categorized into 3 groups: negative (0% positive tumor cells for both CHGA and SYP), low (1% to 9% positive tumor cells for either CHGA or SYP), high (≥10% positive tumor cells for either CHGA or SYP).

**FIGURE 1:**
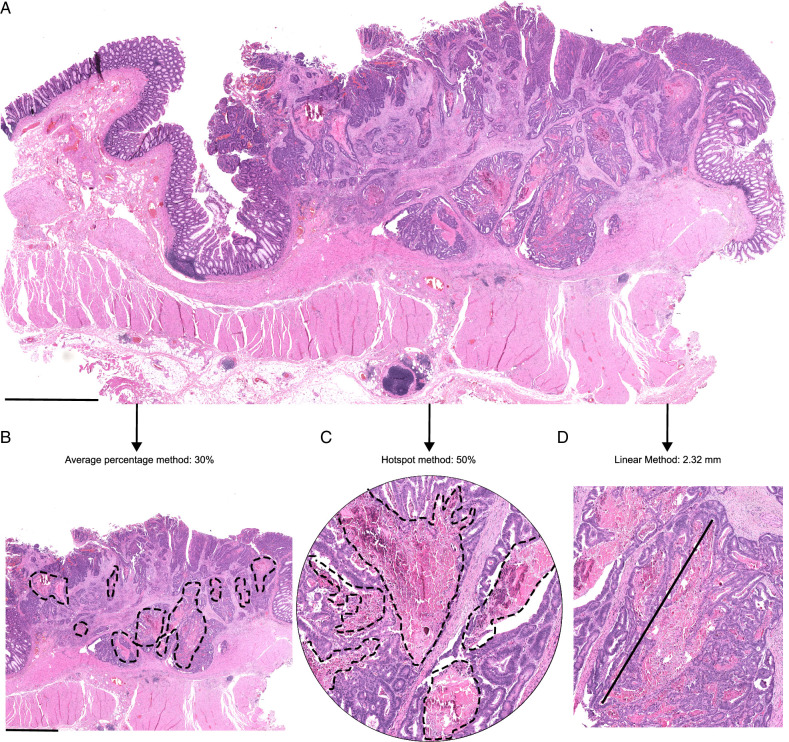
Three evaluation methods for tumor necrosis in colorectal cancer. (A) Overview of a tumor sample. (B) Evaluation of tumor necrosis using the average percentage method. (C) Evaluation of tumor necrosis using the hotspot method. (D) Evaluation of tumor necrosis using the linear method. Scale bars correspond to 2.5 mm.

Tumor necrosis was identified as an area that had nuclear shrinkage, fragmentation, and disappearance, frequently associated with eosinophilia and neutrophil infiltration. A detailed description of the criteria used in its evaluation with example images is included in the Tumor necrosis evaluation manual (Supplementary file 1, Supplemental Digital Content 1, http://links.lww.com/PAS/B909). Tumor necrosis percentage was estimated in cohort 1 cases for a previous study,^4^ but after defining the new criteria, all cases were re-evaluated for this study. Necrosis evaluations were done blinded to the study endpoint. Three necrosis estimation methods were used in both cohorts (Fig. 1). First, in the “average percentage method,” the percentage of tumor necrosis relative to the tumor epithelial area in all available tumor slides was visually evaluated. Second, in the “hotspot method,” a circle with a radius of 1 mm (corresponding to an average field-of-view using a 10x objective magnification in a microscope) was placed in a necrotic hotspot, where the necrotic area would cover the largest possible proportion of circle. The percentage of the necrotic area relative to the total circle area was then visually evaluated. Third, in the “linear method,” the maximum length of a single necrotic region was measured. These evaluations were conducted on digital microscope slides scanned with either Hamamatsu (NanoZoomer S60 or NanoZoomer-XR) or Leica Aperio AT2 slide scanner. An average of 3 digital slides (range, 1 to 18) per case were available for cohort 2, while one digital slide per case, including the deepest tumor invasion, was available for cohort 1. Thirty consecutive cases from cohort 2 were used to investigate the potential influence of slide selection on the results of cohort 1. In these cases, Spearman’s correlation coefficients between tumor necrosis evaluation from a single slide with the deepest tumor invasion and tumor necrosis evaluation using multiple slides were 0.94 for the average percentage method, 0.88 for the hotspot method, and 0.86 for the linear method, indicating high consistency between the results based on analysis of single tumor slide with the deepest invasion vs. multiple slides.

### Statistical Analyses

Statistical analyses were performed with IBM SPSS Statistics for Windows (version 26.0; IBM Corp.). *P* value of <0.05 was considered statistically significant.

Tumor necrosis measurements were classified into 3 categories, following the example of the Immunoscore®^13^ and the ITBCC classification for tumor budding.^7^ The cutoff values were determined based on the previous study,^14^ as well as the shape of the receiver-operating-characteristics (ROC) curves (endpoint: cancer death), where the lowest and highest segments were more concave than the middle segment. The 3 categories for each estimation method were <3%, 3% to 39.9% and ≥40% for the average percentage method, <4%, 4% to 79.9% and ≥80% for the hotspot method, and ≤500 µm, 501 to 3500 µm and >3500 µm for the linear method. Tumor characteristics were cross-tabulated with the 3-category tumor necrosis measurements. The Chi-square test was used to determine the statistical significance.

Colorectal cancer-specific survival was assessed using the Kaplan-Meier method and Cox regression models. The proportional hazards assumption for the Cox regression model was checked using time-dependent variables. Covariates included in the multivariable models were age (<65, 65-75, >75), sex (female, male), stage (I-II, III, IV), tumor budding (grade 1, 2, 3), lymphovascular invasion (no, yes), grade (low-grade, high-grade), year of operation (cohort 1: 2000 to 2005, 2006 to 2010, 2011 to 2015; cohort 2: 2006 to 2010, 2011 to 2015, 2016 to 2020), tumor location (proximal colon, distal colon, rectum), *BRAF* status (wild-type, mutant), and MMR status (proficient, deficient). Cases with missing data (*BRAF* status: 1 patient in cohort 1 and 7 patients in cohort 2) were excluded from the multivariable survival models. For subgroup analysis, 2-category necrosis variables were used: average percentage method, <40% and ≥40%; hotspot method <80% and ≥80%; linear method ≤3500 µm and >3500 µm.

The reproducibility of tumor necrosis evaluation was examined by using Spearman’s correlation coefficients and Cohen’s kappa coefficients. In addition to the 3-category variables, the 2-category variables were included in Cohen’s kappa analysis.

## RESULTS

After excluding patients who received neoadjuvant treatment, 1100 patients were analyzed in cohort 1 and 776 patients in cohort 2. The 3 necrosis evaluation methods mainly showed similar associations with tumor and patient characteristics, including high stage, MMR proficient status, *BRAF* wild-type status, nonmucinous/nonsignet ring cell histology, and high tumor grade (all *P*<0.001) (Table 1; and Table S1, Supplemental Digital Content 2, http://links.lww.com/PAS/B910, Table S2, Supplemental Digital Content 3, http://links.lww.com/PAS/B911). Age, sex, neuroendocrine differentiation, and lymphatic or venous invasion did not have a statistically significant association with any necrosis estimation method. The hotspot method (Table S1, Supplemental Digital Content 2, http://links.lww.com/PAS/B910) and the average percentage method (Table 1) both showed association with distal tumor location in cohorts 1 and 2, while the linear method was not significantly associated with tumor location (*P*=0.238) in cohort 2 (Table S2, Supplemental Digital Content 3, http://links.lww.com/PAS/B911).

**TABLE 1:** Patient and Tumor Characteristics according to Tumor Necrosis Percentage (average necrosis percentage method) in Cohorts 1 and 2

	Cohort 1, N=1100				Cohort 2, N=776			
		Tumor necrosis percentage, N (%)				Tumor necrosis percentage, N (%)		
Variable	Total, N (%)	<3%	3-39.9%	≥40%	Total, N (%)	<3%	3-39.9%	≥40%
Sex								
Male	557 (51%)	122 (22%)	399 (72%)	36 (6.5%)	412 (53%)	116 (28%)	271 (66%)	25 (6.1%)
Female	543 (49%)	131 (24%)	376 (69%)	36 (6.6%)	364 (47%)	119 (33%)	216 (59%)	29 (8.0%)
*P*		0.662				0.166		
Age								
<65	290 (26%)	59 (20%)	212 (73%)	19 (6.6%)	233 (30%)	66 (28%)	154 (66%)	13 (5.6%)
65-75	381 (35%)	82 (22%)	272 (71%)	27 (7.1%)	285 (37%)	88 (31%)	174 (61%)	23 (8.1%)
>75	429 (39%)	112 (26%)	291 (68%)	26 (6.1%)	258 (33%)	81 (31%)	159 (62%)	18 (7.0%)
*P*		0.389				0.704		
Tumor location								
Proximal colon	536 (49%)	151 (28%)	354 (66%)	31 (5.8%)	323 (42%)	110 (34%)	190 (59%)	23 (7.1%)
Distal colon	404 (37%)	62 (15%)	306 (76%)	36 (8.9%)	205 (26%)	41 (20%)	148 (72%)	16 (7.8%)
Rectum	160 (15%)	40 (25%)	115 (72%)	5 (3.1%)	248 (32%)	84 (34%)	149 (60%)	15 (6.0%)
*P*		<0.001				0.007		
Stage								
I	184 (17%)	62 (34%)	120 (65%)	2 (1.1%)	187 (24%)	89 (48%)	97 (52%)	1 (0.5%)
II	408 (37%)	83 (20%)	293 (72%)	32 (7.8%)	253 (33%)	71 (28%)	157 (62%)	25 (9.9%)
III	355 (32%)	77 (22%)	258 (73%)	20 (5.6%)	251 (32%)	64 (25%)	170 (68%)	17 (6.8%)
IV	153 (14%)	31 (20%)	104 (68%)	18 (12%)	85 (11%)	11 (13%)	63 (74%)	11 (13%)
*P*		<0.001				<0.001		
Histologic subtype								
Adenocarcinoma	995 (90%)	201 (20%)	725 (73%)	69 (6.9%)	700 (90%)	188 (27%)	458 (65%)	54 (7.7%)
Mucinous carcinoma	77 (7.0%)	39 (51%)	36 (47%)	2 (2.6%)	61 (7.9%)	39 (64%)	22 (36%)	0 (0%)
Signet ring cell carcinoma	28 (2.5%)	13 (46%)	14 (50%)	1 (3.6%)	15 (1.9%)	8 (53%)	7 (47%)	0 (0%)
*P*		<0.001				<0.001		
Neuroendocrine differentiation*								
0%	—	—	—	—	560 (72%)	162 (29%)	358 (64%)	40 (7.1%)
1–9%	—	—	—	—	157 (20%)	47 (30%)	98 (62%)	12 (7.6%)
≥10%	—	—	—	—	43 (5.5%)	15 (35%)	26 (60%)	2 (4.7%)
Missing data					16 (2.1%)			
*P*						0.905		
WHO grade								
Low-grade	903 (82%)	188 (21%)	662 (73%)	53 (5.9%)	665 (86%)	197 (30%)	429 (64%)	40 (6.0%)
High-grade	197 (18%)	65 (33%)	113 (57%)	19 (19%)	111 (14%)	38 (34%)	59 (53%)	14 (13%)
*P*		<0.001				0.011		
Lymphovascular invasion								
No	858 (78%)	202 (24%)	596 (69%)	60 (7.0%)	429 (55%)	148 (34%)	258 (60%)	23 (5.4%)
Yes	242 (22%)	51 (21%)	179 (74%)	12 (5.0%)	347 (45%)	87 (25%)	229 (66%)	31 (8.9%)
*P*		0.327				0.006		
Mismatch repair status								
MMR proficient	931 (85%)	188 (20%)	684 (73%)	59 (6.3%)	652 (84%)	170 (26%)	439 (67%)	43 (6.6%)
MMR deficient	169 (15%)	65 (38%)	91 (54%)	13 (7.7%)	124 (16%)	65 (52%)	48 (39%)	11 (8.9%)
*P*		<0.001				<0.001		
*BRAF* status								
Wild-type	916 (83%)	187 (20%)	668 (73%)	61 (6.7%)	662 (85%)	174 (26%)	445 (67%)	43 (6.5%)
Mutant	182 (17%)	65 (36%)	106 (58%)	11 (6.0%)	107 (14%)	55 (51%)	41 (38%)	11 (10%)
Missing data	2 (0.2%)				7 (0.9%)			
*P*		<0.001				<0.001		

* Neuroendocrine differentiation was determined for cohort 2 using synaptophysin and chromogranin A immunohistochemistry.

— indicates missing data for neuroendocrine differentiation in cohort 1.

MMR indicates mismatch repair.

**FIGURE 2:**
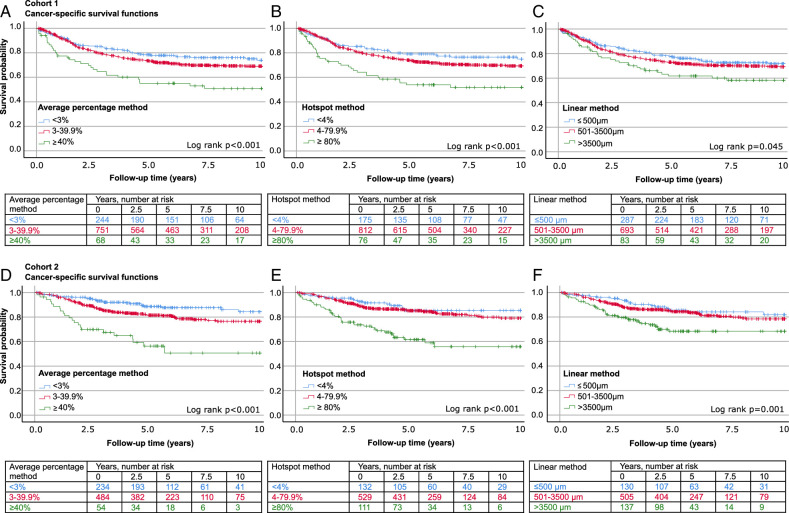
Tumor necrosis evaluation methods and survival. Kaplan-Meier cancer-specific survival curves for the 3 tumor necrosis evaluation methods in cohort 1 (A-C) and cohort 2 (D-F).

In ROC analysis, the average percentage and hotspot methods had a slightly higher area under the curve (AUC) in predicting patients with colorectal cancer-specific mortality than the linear method, but the difference was within the limits of 95% CIs (Figure S1, Supplemental Digital Content 4, http://links.lww.com/PAS/B912). As categorical variables, all 3 methods reached statistical significance in Kaplan-Meier analysis (Fig. 2), univariable Cox regression models, and multivariable Cox regression models (Table 2; and Table S3, Supplemental Digital Content 5, http://links.lww.com/PAS/B913; Table S4, Supplemental Digital Content 6, http://links.lww.com/PAS/B914; and Table S5, Supplemental Digital Content 7, http://links.lww.com/PAS/B915). The survival substantially declined in the category with the highest necrosis (≥40%, ≥80%, or >3500 µm) compared to the 2 necrosis categories with the least amount of tumor necrosis (Fig. 2). The greatest hazard ratios were observed in both univariable and multivariable models for the average necrosis percentage ≥40% vs. <3% (univariable cohort 1: HR 2.41; 95% CI, 1.55-3.74 and cohort 2: HR 4.85; 95% CI, 2.73-8.16; multivariable cohort 1: HR 3.03; 95% CI, 1.93-4.78, cohort 2: HR 2.97; 95% CI, 1.63-5.40) (Table 2, and Table S3, Supplemental Digital Content 5, http://links.lww.com/PAS/B913).

**TABLE 2:** Cox Proportional Hazards Regression Models for the Associations between Tumor Necrosis Evaluation Methods and Colorectal Cancer-specific Survival in Cohorts 1 and 2

	Cohort 1, N=1063				Cohort 2, N=771			
Variable	No. of cases	No. of events	Univariable HR (95% CI)	Multivariable HR (95% CI)	No. of cases	No. of events	Univariable HR (95% CI)	Multivariable HR (95% CI)
Average percentage method								
<3%	244	55	1 (referent)	1 (referent)	233	25	1 (referent)	1 (referent)
3-39.9%	751	210	1.26 (0.94-1.70)	1.47 (1.07-2.00)	484	88	1.75 (1.12-2.73)	1.11 (0.71-1.74)
≥40%	68	31	2.41 (1.55-3.74)	3.03 (1.93-4.78)	54	22	4.85 (2.73-8.16)	2.97 (1.63-5.40)
*P*_trend_			<0.001	<0.001			<0.001	0.003
Hotspot method								
<4%	175	38	1 (referent)	1 (referent)	131	15	1 (referent)	1 (referent)
4-79.9%	812	224	1.28 (0.91-1.80)	1.39 (0.97-2.00)	529	81	1.34 (0.77-2.32)	0.84 (0.47-1.51)
≥80%	76	34	2.51 (1.58-3.98)	2.76 (1.72-4.24)	111	39	3.80 (2.09-6.90)	1.82 (0.96-3.44)
*P*_trend_			<0.001	<0.001			<0.001	0.004
Linear method								
≤500 μm	287	71	1 (referent)	1 (referent)	129	17	1 (referent)	1 (referent)
501-3500 μm	693	193	1.15 (0.88-1.51)	1.36 (1.02-1.80)	505	81	1.27 (0.75-2.15)	1.24 (0.72-2.13)
>3500 μm	83	32	1.69 (1.11-2.57)	2.06 (1.34-3.17)	137	37	2.50 (1.40-4.44)	1.96 (1.06-3.62)
*P*_trend_			0.029	0.001			0.001	0.016

Cox proportional hazards regression model was adjusted for age (<65, 65-75, >75), sex (female, male), stage (I-II, III, IV), tumor budding (grades 1, 2, 3), lymphovascular invasion (no, yes), grade (low-grade, high-grade), year of operation (cohort 1: 2000-2005, 2006-2010, 2011-2015; Cohort 2: 2006-2010, 2011-2015, 2016-2020), tumor location (proximal colon, distal colon, rectum), *BRAF* status (wild-type, mutant) and mismatch repair status (proficient, deficient). Cases with missing data (*BRAF* status: 1 patient in cohort 1 and 7 patients in cohort 2) were excluded from the multivariable survival models. CI indicates confidence interval; HR, hazards ratio.

Considering that most tumors were MMR proficient (cohort 1, 85%; cohort 2, 84%), we conducted additional sensitivity analysis limited to MMR proficient cases. Kaplan-Meier analyses showed statistically significant survival associations for the average percentage method and hotspot method in both cohorts (Figure S2, Supplemental Digital Content 8, http://links.lww.com/PAS/B916). In multivariable Cox regression models, all 3 methods showed statistically significant association with survival, and the average percentage method (≥40% vs. <3%) showed the greatest HRs (cohort 1: HR 2.60; 95% CI, 1.58-4.29; cohort 2: HR 3.42; 95% CI, 1.78-6.55) (Table S6, Supplemental Digital Content 9, http://links.lww.com/PAS/B915).

Additional subgroup analyses of the prognostic value of tumor necrosis are presented as forest plots (Figure S3, Supplemental Digital Content 10, http://links.lww.com/PAS/B917). In these analyses, tumor necrosis predicted mortality in a wide range of patient subgroups defined by clinical, pathologic, and molecular characteristics. Although tumor necrosis appeared to show stronger prognostic significance in females (linear method) and lower T stages (hotspot method and linear method) in cohort 1 and patients with low-grade tumors (average percentage method and hotspot method) in cohort 2, no consistent findings of a statistically significant difference according to any clinicopathologic feature were observed across both cohorts.

**FIGURE 3:**
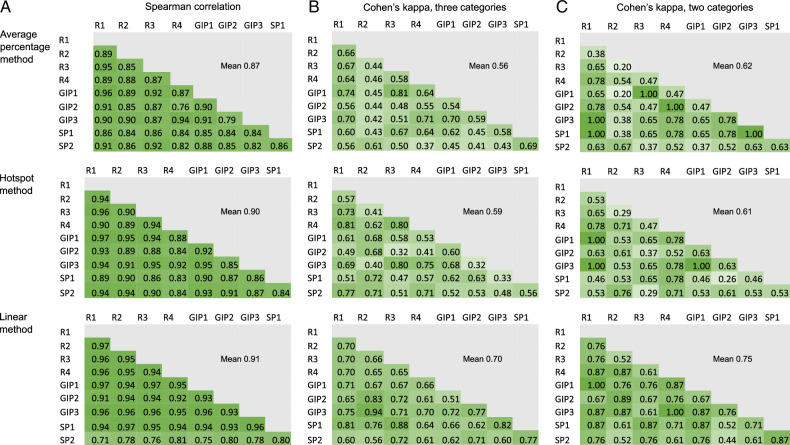
Reproducibility analysis for the 3 tumor necrosis estimation methods. The reproducibility was measured with Spearman rank correlation analysis (A) and Cohen’s kappa (B–C).GIP, gastrointestinal pathologist; R, researcher; SP, specializing pathologist.

The reproducibility of the 3 necrosis estimation methods was tested (Fig. 3). Tumor necrosis was assessed from 30 tumor samples by 3 gastrointestinal pathologists, 2 specializing pathologists, and 4 researchers. The mean Spearman’s correlation coefficient for the average necrosis percentage was 0.87, for the hotspot method 0.90, and the linear measure 0.91, indicating excellent reproducibility and minimal difference between the estimation methods. However, when categorized into 3-category variables or 2-category variables, the linear method reached slightly higher mean Cohen’s kappa than the other methods (3-category: average necrosis percentage 0.56, hotspot method 0.59, linear method 0.70; 2-category: mean necrosis percentage 0.62, hotspot method 0.61, linear method 0.75) (Fig. 3). These results suggest that the linear method had slightly higher accuracy in measuring cases near category cut-points than the other 2 methods.

## DISCUSSION

Our objective was to define an accurate and reproducible tumor necrosis evaluation method to enable its routine application as a prognostic parameter in CRC. Two large cohorts consisting of more than 1800 patients were analyzed with 3 necrosis estimation methods (average necrosis percentage, necrosis percentage in a single hotspot, and the largest diameter of a single necrotic focus). All 3 methods showed prognostic value independent of other tumor and patient characteristics, as well as reasonably high reproducibility.

Several previous colorectal cancer studies^5,6,15,16^ have evaluated tumor necrosis as the percentage of the necrotic area of the total tumor area, classified as none, ≤10%, 10% to 30%, and ≥30%. Richards et al.^5^ tested the reproducibility of this evaluation between 2 researchers, the interobserver intraclass correlation coefficient was 0.86. This evaluation method has also been used for upper urinary tract cell carcinoma^17^ and lung cancer.^15^ In non-small cell lung cancer, reproducibility kappa was 0.8 between 2 researchers.^15^ In some studies, necrosis percentage was also classified into 2 categories, such as absent/present^18^ or little (<10%)/abundant (≥10%) necrosis.^19,20^ A more subjective 3-grade assessment (rare areas of necrosis, frequent small areas of necrosis and broad areas of necrosis) has also been applied.^21^ Previous studies have used different cutoff values and various numbers of categories, which makes it difficult to compare different studies with each other.

We hypothesized that the hotspot method and the linear method could have better reproducibility than estimating the total necrosis percentage, as the measurements based on a single hotspot may be easier to reproduce than evaluations based on the whole tumor. However, all 3 methods reached reasonably high reproducibility, with the linear method showing the highest kappa coefficients, when evaluated as a categorical variable. Nevertheless, the average percentage method seemed to slightly outperform the linear method in predicting survival. This may be related to the linear measure being affected by the shape of the necrotic area (high values for long but thin foci). Based on our results, all 3 evaluation methods for tumor necrosis appear to show high reproducibility and prognostic value, and it is difficult to recommend any of the 3 methods over the other 2.

Tumor necrosis is associated with poor differentiation, advanced stage, and more aggressive tumor behavior.^4,14,22^ The factors contributing to the adverse prognostic effect of tumor necrosis are not clear, but some hypotheses can be formed. Necrosis is a form of unregulated cell death where the cell membranes rupture, releasing the contents of the cell and eliciting an inflammatory response.^23,24^ Large amounts of necrosis are associated with systemic inflammation and weaker local inflammatory response.^5,25^ Tumor necrosis has been related to elevated serum levels of proinflammatory cytokines and tumor aggression.^4,26,27^ Consequently, the presence of necrosis may influence the tumor microenvironment by the release of damage-associated molecular patterns (DAMPs) and proinflammatory cytokines, thus leading to more aggressive cancer.^6,23^ Furthermore, necrosis may be a sign or sequel of activated proliferation and anti-apoptotic pathways in tumors which are hallmarks of cancer progression.^28^ Necrosis has been hypothesized to be associated with insufficient blood flow to tumors and hypoxia but there are studies for and against this.^14,26,29^ The formation of tumor necrosis is likely dependent on many factors which could explain the adverse effects related to abundant necrosis.^14,29^

Other promising prognostic markers in CRC include tumor budding,^7^ Immunoscore®,^13^ modified Glasgow prognostic score (mGPS),^30^, and tumor stroma ratio,^31^ among others. Of these prognostic markers, tumor budding is widely used in clinical practice.^3^ In addition to its use in identifying high-risk stage II CRC patients for the consideration of adjuvant therapy, tumor budding has proven a useful marker in identifying high-risk pT1 tumors after endoscopic or transanal tumor removal.^3^ In our study, tumor necrosis had prognostic value independent of tumor budding, disease stage, and tumor molecular features including MMR status and *BRAF* mutation status. Similar to tumor budding, it is easy to evaluate tumor necrosis from H&E-stained samples making it an accessible prognostic marker. Our study defines criteria for tumor necrosis evaluation that can be used to assess whether stage II CRC patients with necrotic tumors would benefit from adjuvant treatment or if tumor necrosis could guide adjuvant treatment options for stage III patients.

The limitations of our study must be considered. First, when sampling tumors, pathologists may avoid overtly necrotic regions, as these are not useful for evaluating tumor morphology or conducting molecular analyses. This may have reduced the average necrosis percentage observed in the tumors. Therefore, it should be studied whether systematic sampling of the most necrotic regions could influence the survival results. Second, the analyses of cohort 1 were based on a single tumor section with the deepest invasion. However, a high correlation was observed between tumor necrosis measurements from a single tumor section with the deepest invasion and measurements from multiple tumor sections. Third, although the study included 2 large cohorts, the necrosis evaluation was based on a retrospective analysis of the tumor samples. A prospective study could assess whether tumor necrosis evaluation could identify patients who would benefit from standard adjuvant treatments or anti-angiogenesis treatments such as anti-VEGF antibody therapies. Fourth, the relevance of tumor necrosis in patients with neoadjuvant-treated rectal cancer is unclear, and it has not been investigated whether tumor necrosis evaluation could be done from preoperative biopsies. Fifth, although the cohorts were large, the number of MMR-deficient cases was relatively low, making the survival estimates less accurate in that patient subgroup. Sixth, these analyses focus mainly on the prognostic relevance of tumor necrosis in resected tumors, while largely excluding primarily metastatic cases that are treated with systemic chemotherapy only.

## CONCLUSIONS

Tumor necrosis can be reproducibly evaluated on H&E-stained sections, which makes it an accessible prognostic factor. All 3 estimation methods predicted patient survival independently of other prognostic parameters and had reasonably high reproducibility. The findings pave the way for the use of tumor necrosis as a prognostic factor in colorectal cancer.

## Supplementary Material

SUPPLEMENTARY MATERIAL
