# Supplementary material for: Establishing Criteria for Tumor Necrosis as Prognostic Indicator in Colorectal Cancer
Source: Am J Surg Pathol. 2024 Jul 15;48(10):1284–92. doi: 10.1097/PAS.0000000000002286 (PMC11404753; doi:10.1097/PAS.0000000000002286)

Kastinen M, et al. Establishing criteria for tumor necrosis as prognostic indicator in colorectal cancer. Supplementary file 1.

Tumor necrosis evaluation manual:

Criteria for tumor necrosis evaluation in colorectal cancer

Contents

1 Definition and criteria for tumor necrosis 2

Special considerations in tumor necrosis evaluation: mucinous tumors 4

Special considerations in tumor necrosis evaluation: excluded regions from tumor necrosis evaluation 5

2 Tumor necrosis evaluation methods 6

Average percentage method 7

Linear method 8

Hotspot method 9

3 Example cases 11

Example 1 11

Example 2 13

Example 3 15

Example 4 17

1 Definition and criteria for tumor necrosis

Necrosis is a form of uncontrolled cell death associated with the following features in hematoxylin & eosin (H&E) -stained histology slides

- Shadows of tumor cells
- Nuclear shrinkage, karyorrhexis, karyolysis
- Areas can have increased eosinophilia
- Neutrophils are often present in necrotic tissue

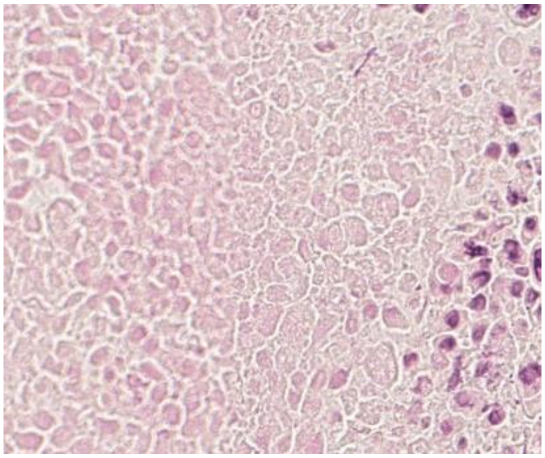

Schematic representation of the phases of tumor cell necrosis

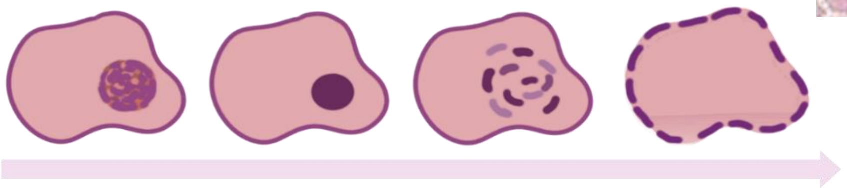

Non-necrotic tumor cell

Pyknosis

Nucleus shrinks

Karyorrhexis

Nucleus is fragmented

Karyolysis

Nucleus diseappears, and a shadow of the tumor cell remains.

Necrosis in tumor resections can have various of appearances, all of which are included in the necrosis evaluation

- Intraluminal necrosis, i.e., necrosis within glandular lumina
- Large necrotic areas extending to surrounding tissue. These frequently show collagen strands among the necrotic tissue

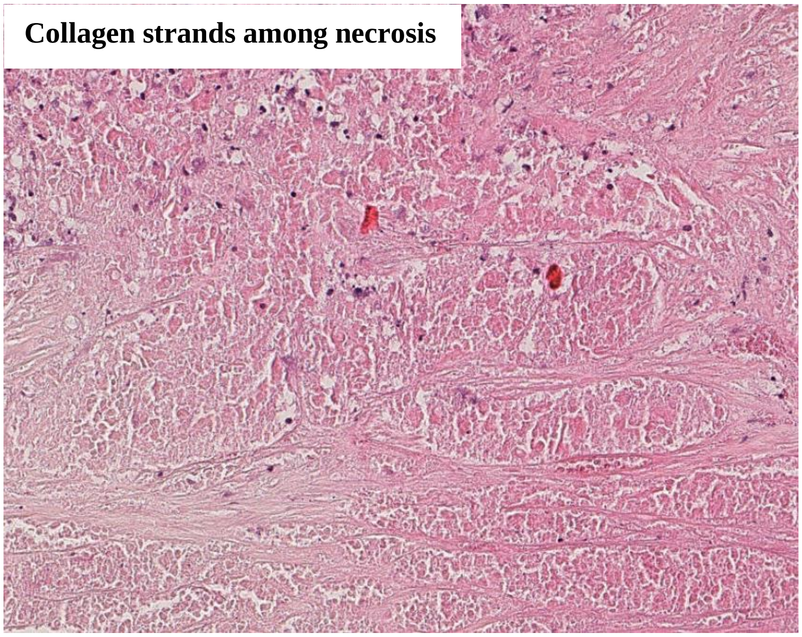

Collagen strands among necrosis

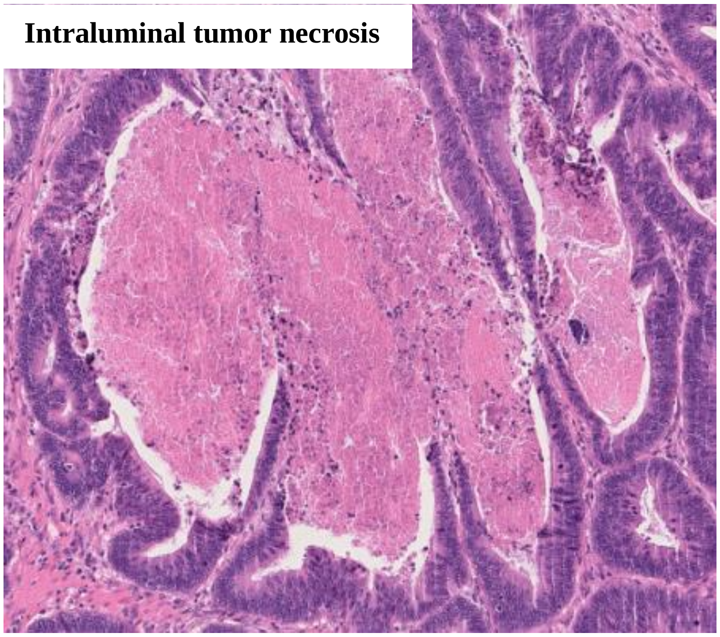

Intraluminal tumor necrosis

Example of a tumor showing large numbers of neutrophils in the necrotic tumor tissue.

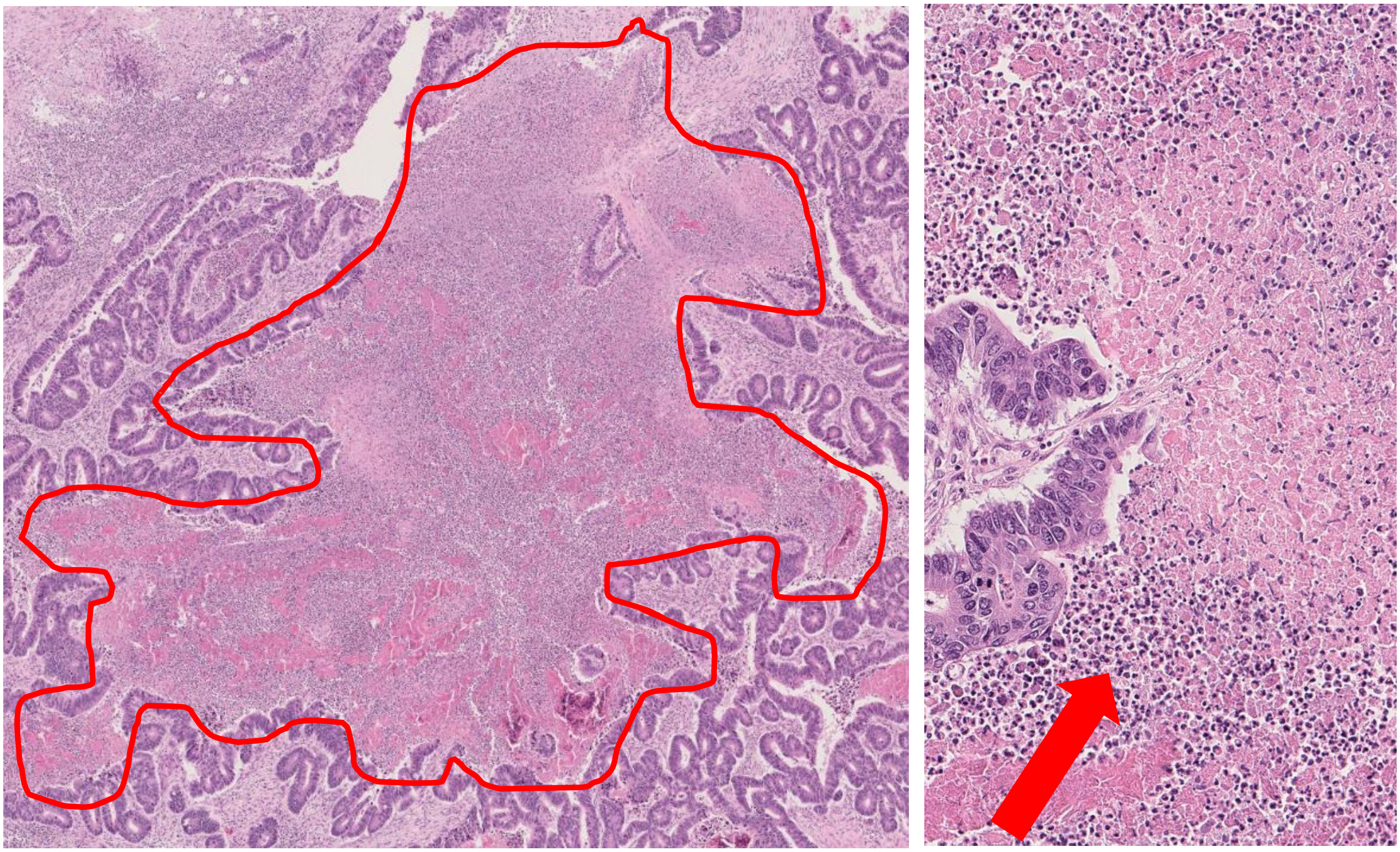

Special considerations in tumor necrosis evaluation: mucinous tumors

It can be difficult to evaluate tumor necrosis in mucinous tumors, as necrotic cells spread out in mucin. Necrotic tumor cells within mucinous regions of the tumors are detected based on the same criteria as those in non-mucinous regions and are included in tumor necrosis evaluation.

Examples of necrotic cells within mucin:

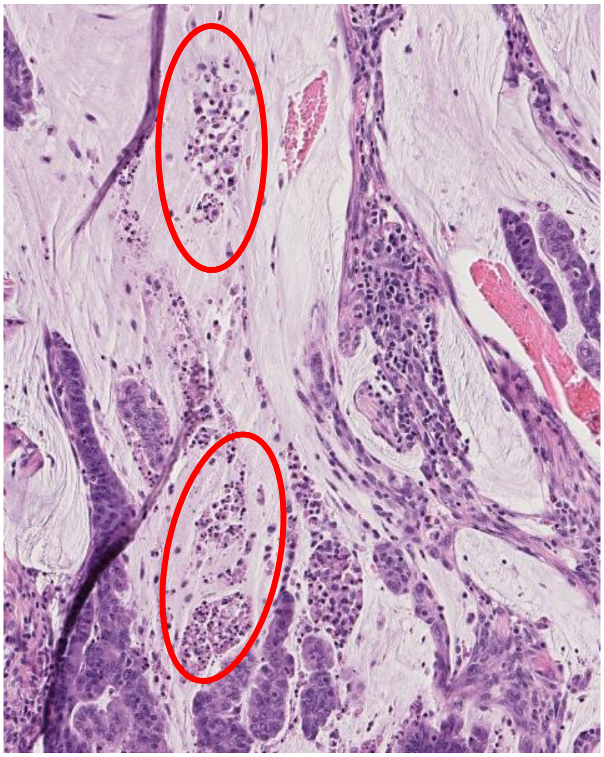

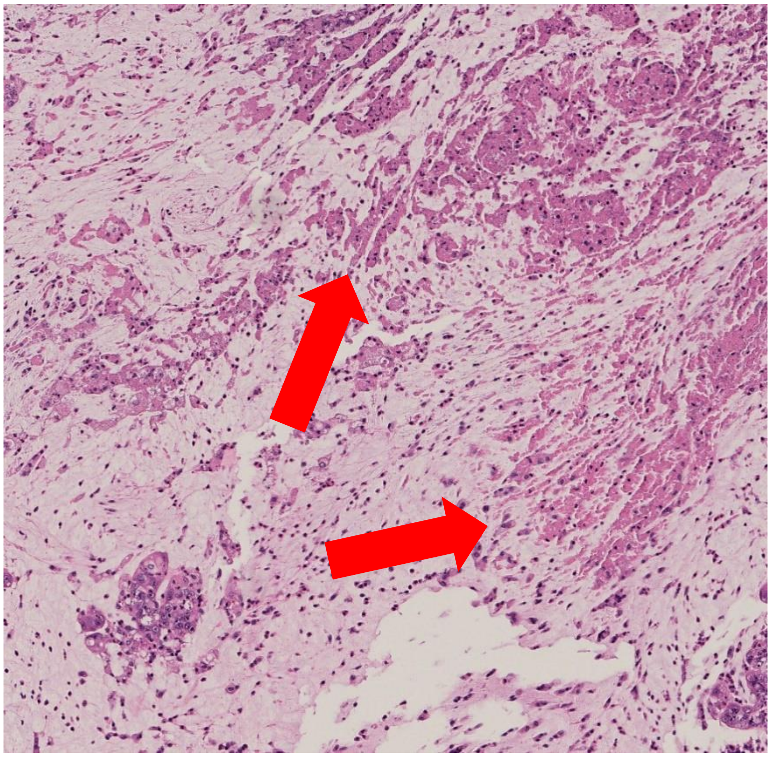

Special considerations in tumor necrosis evaluation: excluded regions from tumor necrosis evaluation

The following regions should not be included in the evaluation of tumor necrosis in colorectal cancer:

- Lymph node metastases
- Tumor necrosis associated with ulcerated tumor surface
  - Tumor necrosis should not have direct contact to the surface of the tumor
- Neutrophilic abscesses within colorectal tumors that are not associated with tumor cell necrosis. These are most often seen in the tumor invasive margin.

Example of tumor necrosis associated with surface ulceration that should be excluded from the evaluation

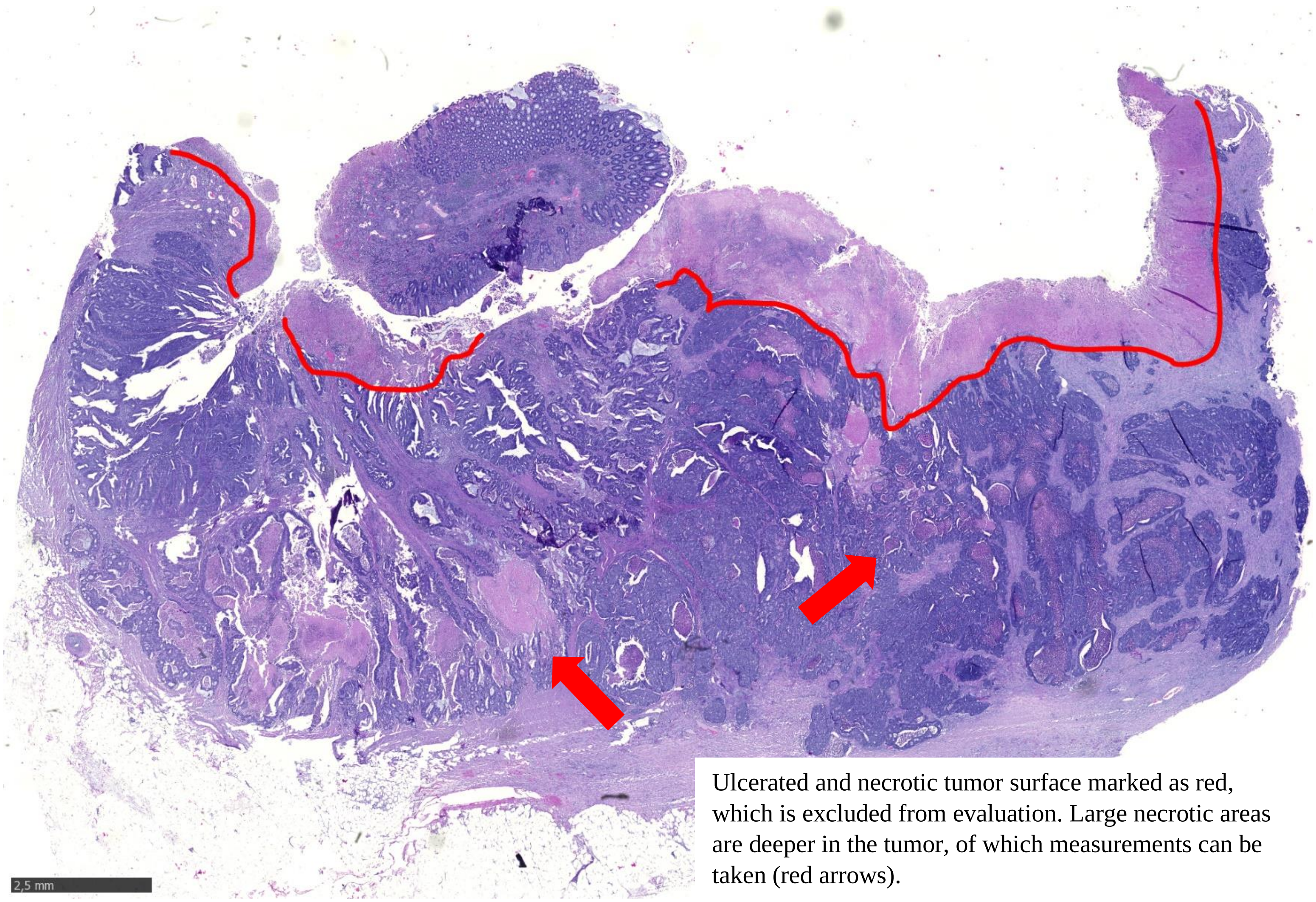

Ulcerated and necrotic tumor surface marked as red, which is excluded from evaluation. Large necrotic areas are deeper in the tumor, of which measurements can be taken (red arrows).

2 Tumor necrosis evaluation methods

Three methods for tumor necrosis evaluation:

1. **Average percentage method**: percentage of necrotic tumor tissue area relative to total tumor epithelial area
2. **Linear method**: length of a single largest necrotic focus
3. **Hotspot method**: percentage of tumor necrosis in a necrotic hotspot (circle of 2.0 mm diameter), where the necrotic area covers the largest possible area of the hotspot

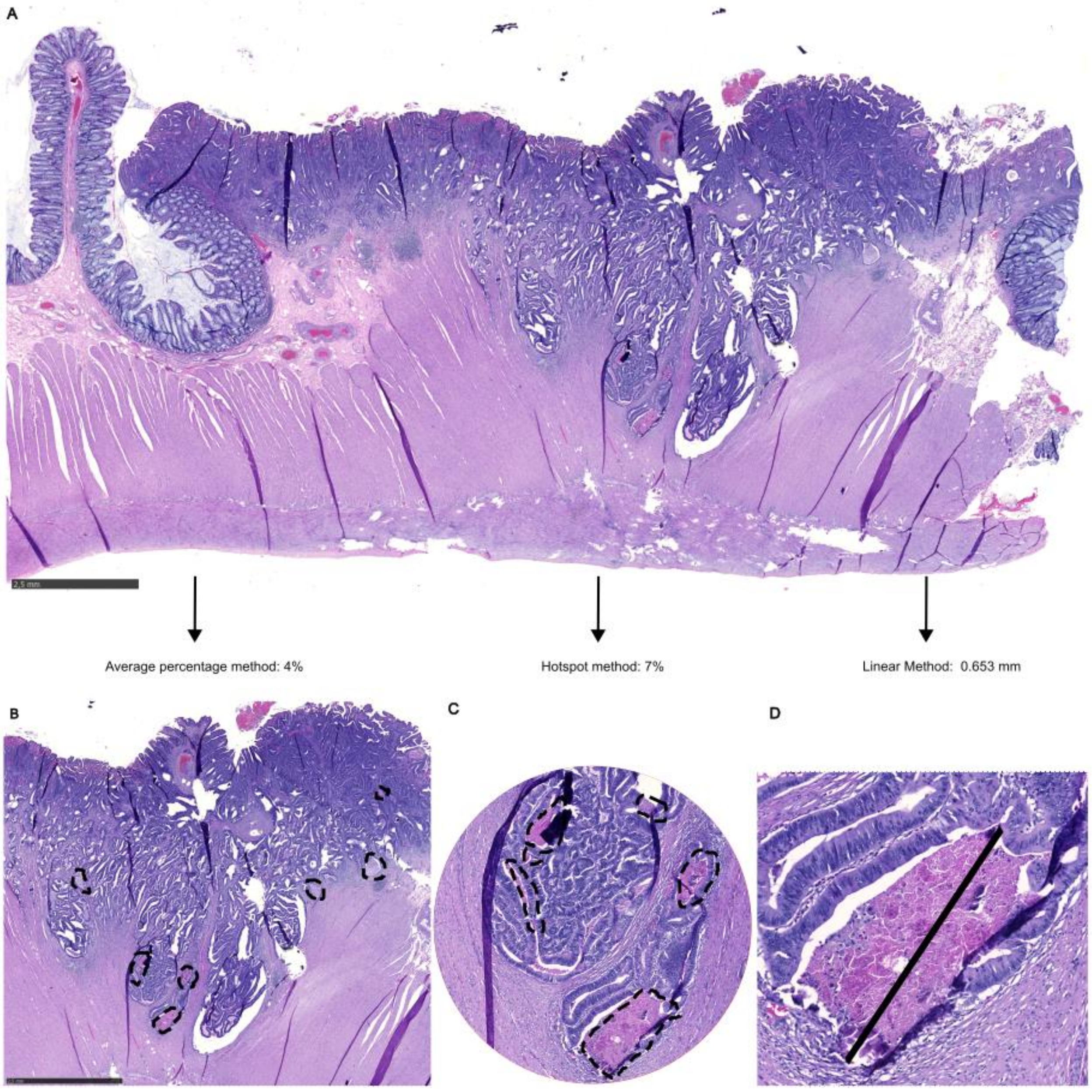

Average percentage method

Definition: percentage of necrotic tumor tissue area relative to total tumor epithelial area

- Based on visual examination of all available tumor slides
- Percentages of 0 to 10 are evaluated in one percent accuracy, and percentages of 10 to 100 in five percent accuracy: 15%, 20%, 25%, etc.
- The percentages can be categorized into the following categories
  - Low: <3%
  - Intermediate: 3-39.9%
  - High: ≥40%

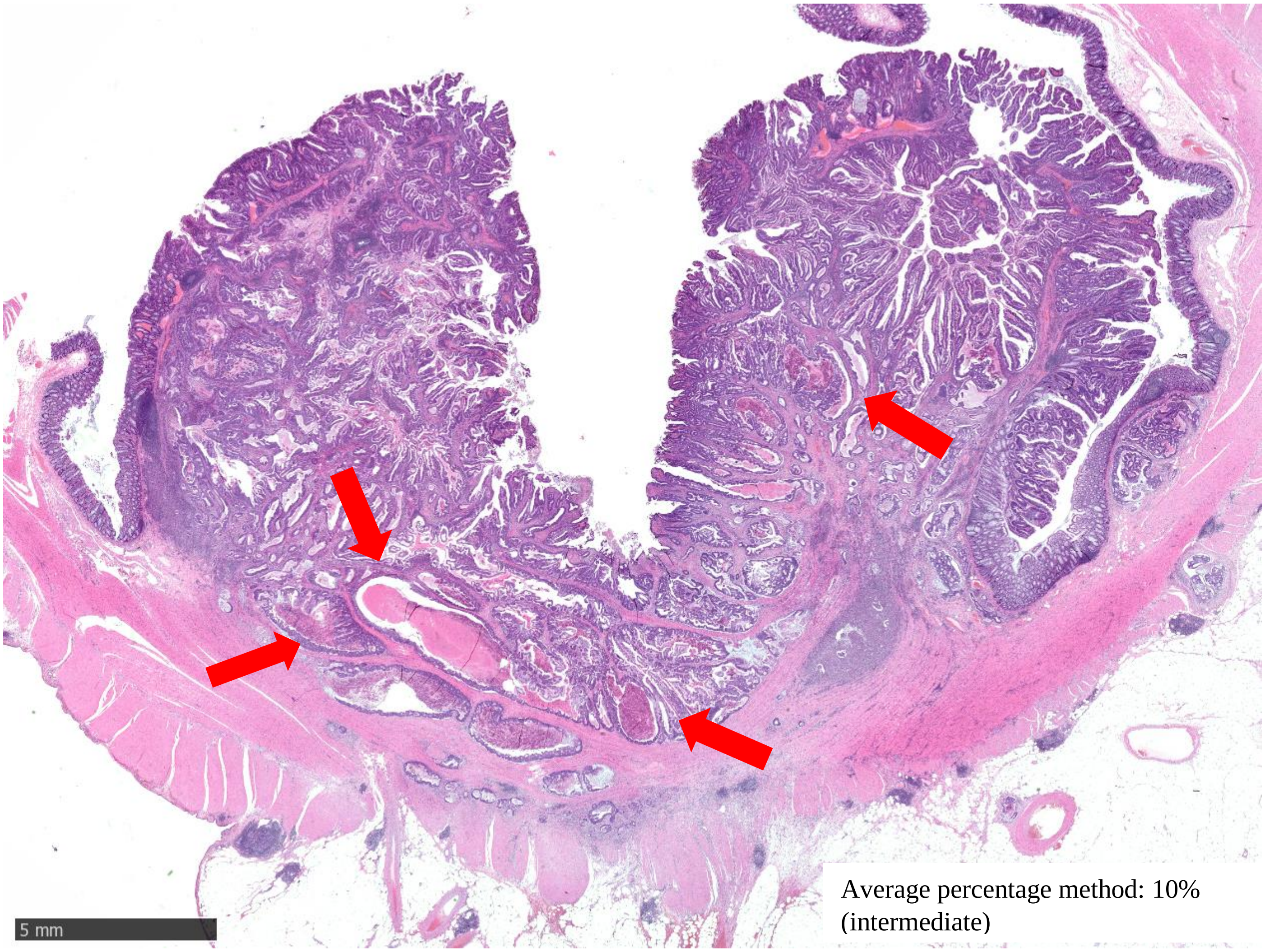

Average percentage method: 10% (intermediate)

Linear method

Definition: Length of a single largest necrotic focus in a tumor

- Linear measure in µm should be taken from a single hotspot that has the longest united region of tumor necrosis in all available tumor slides
- The measurements can be categorized into the following categories
    - Low: ≤500 µm
    - Intermediate: 501-3500 µm
    - High: >3500 µm

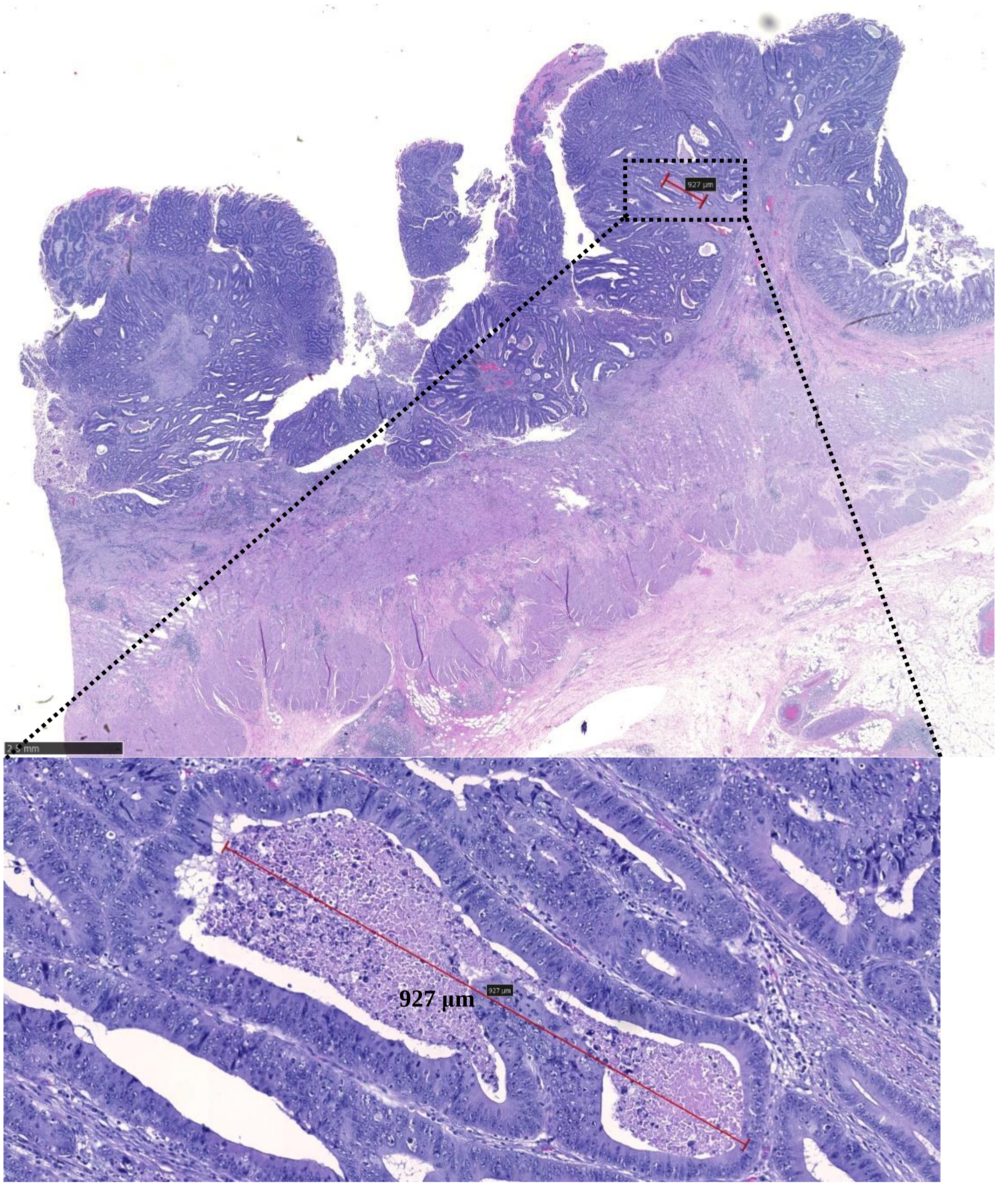

Hotspot method

Definition: percentage of tumor necrosis in a necrotic hotspot (circle of 2.0 mm diameter), where the necrotic area covers the largest possible area of the hotspot

- Percentages of 0 to 10 are evaluated in one percent accuracy, and percentages of 10 to 100 in five percent accuracy: 15%, 20%, 25%, etc.
- The circle corresponds to a 10x (objective magnification) field of view, when using a conventional microscope with 20 mm eyepieces.
- The percentages can be categorized into the following categories
    - Low: <4%
    - Intermediate: 4-79.9%
    - High: ≥80%

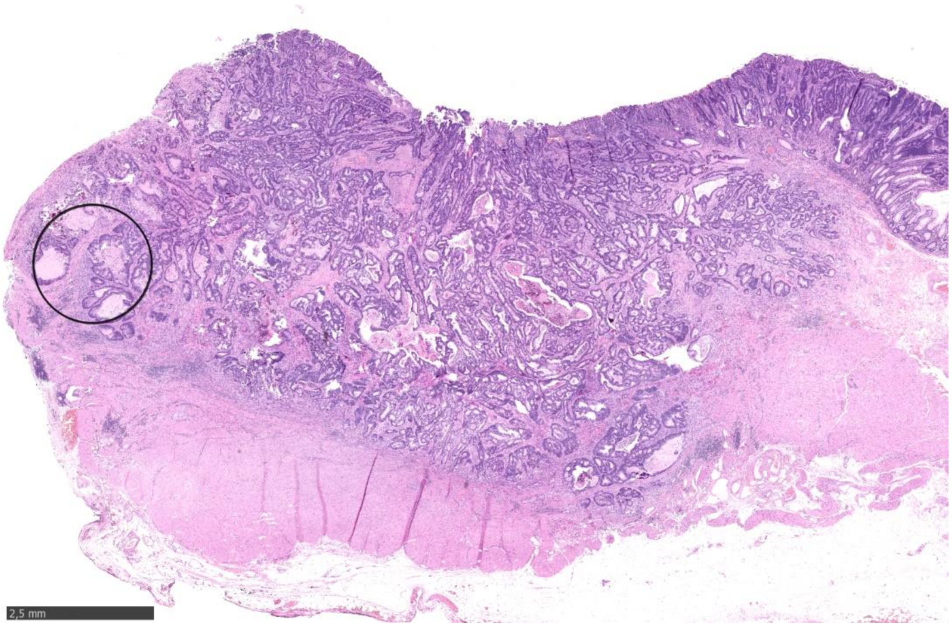

Hotspot method: 25% (intermediate)

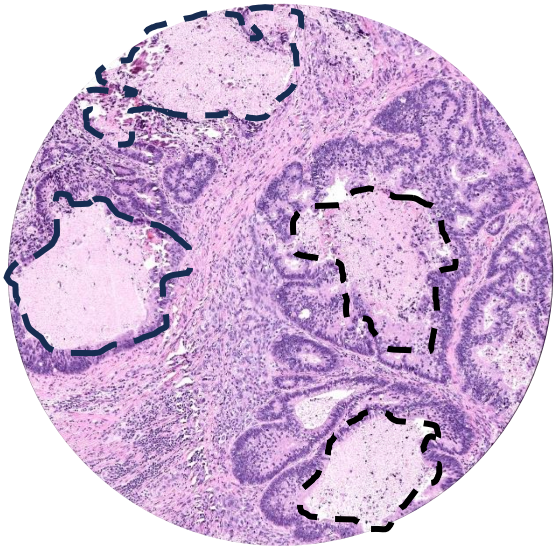

Examples of tumor necrosis hotspots with various tumor necrosis percentages:

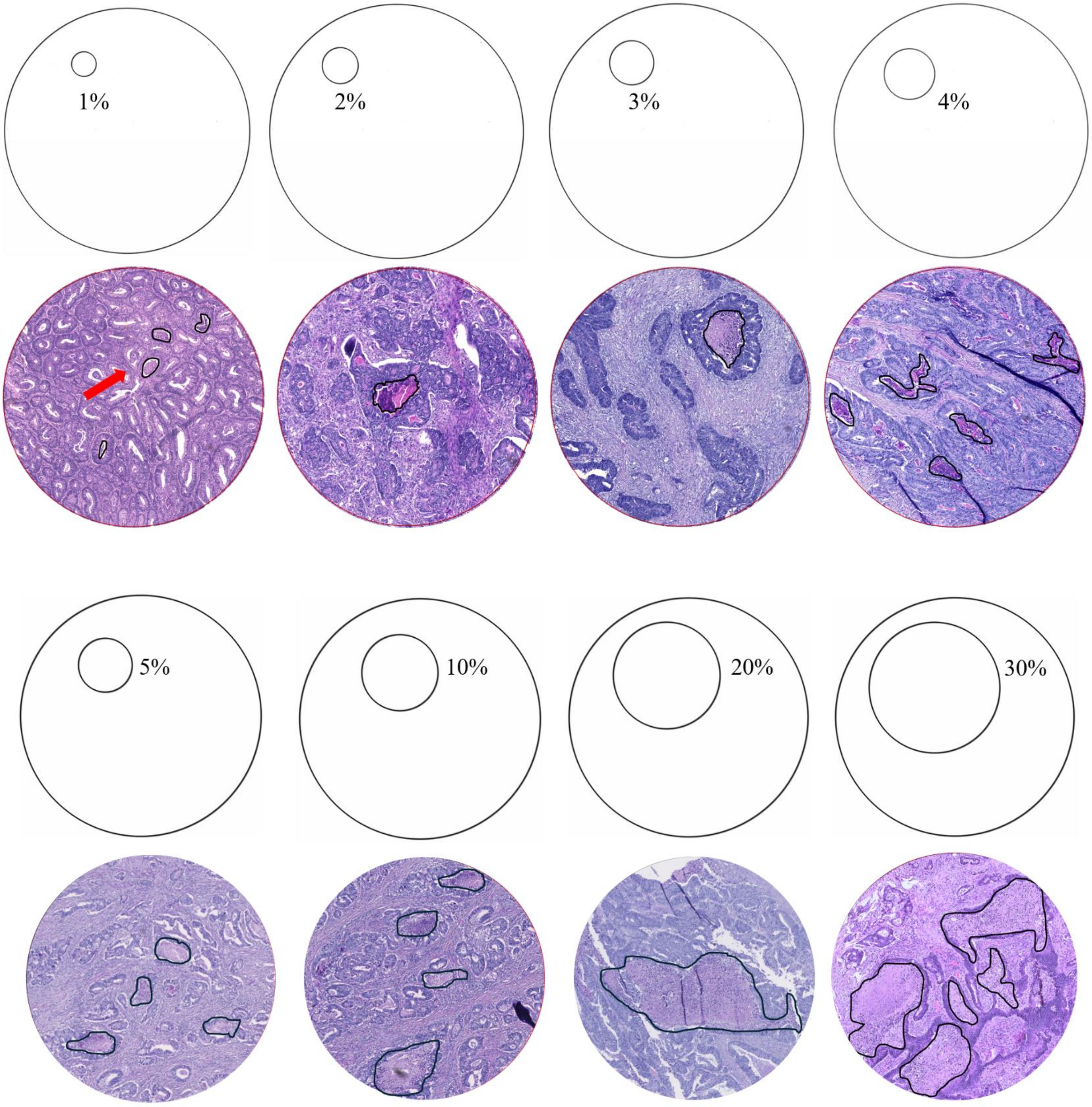

3 Example cases

Example 1

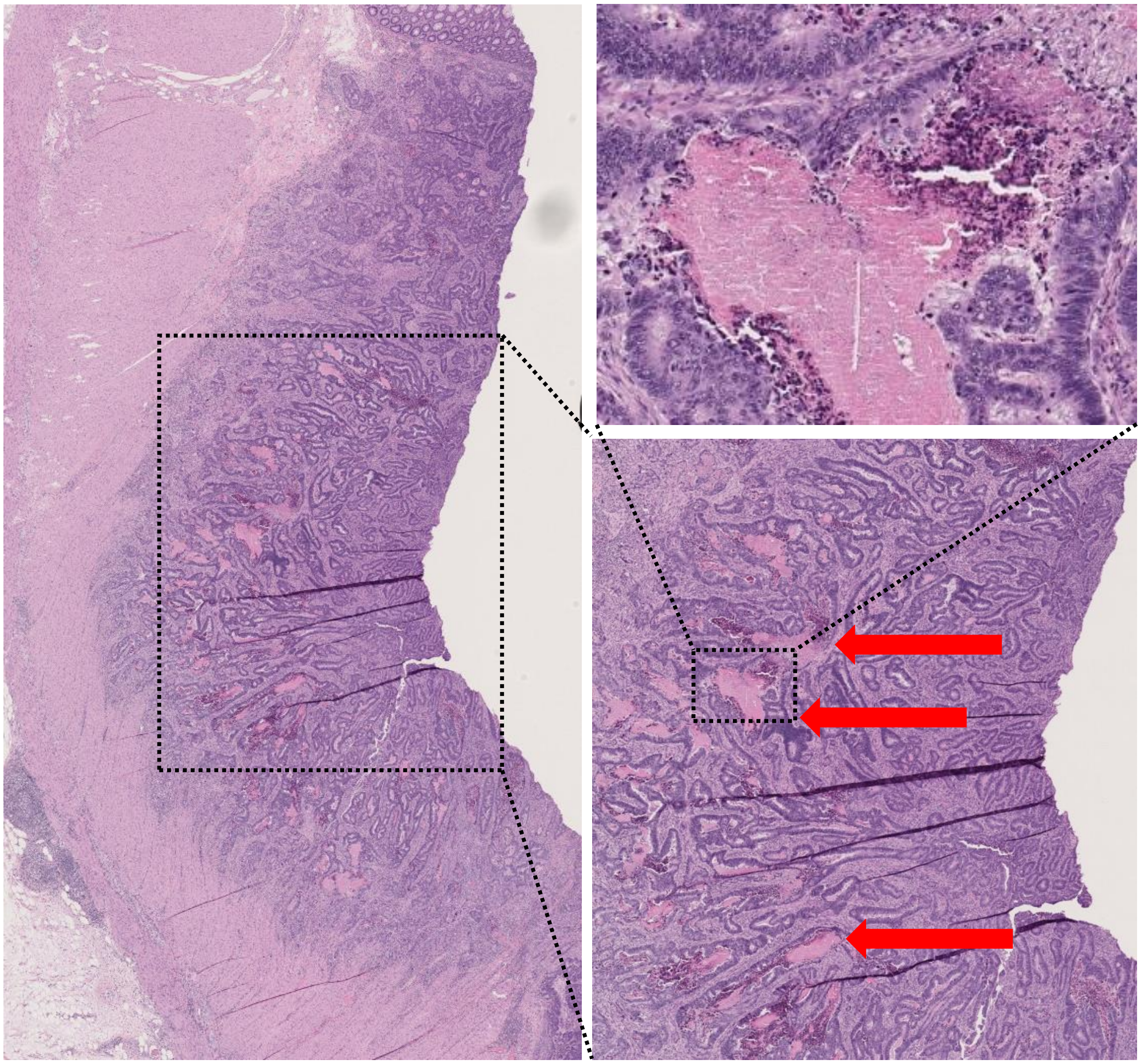

Tumor necrosis evaluation example 1

- Average percentage method: 10% (intermediate)
- Hotspot method: 20% (intermediate)
- Linear method: 1410 μm (intermediate)

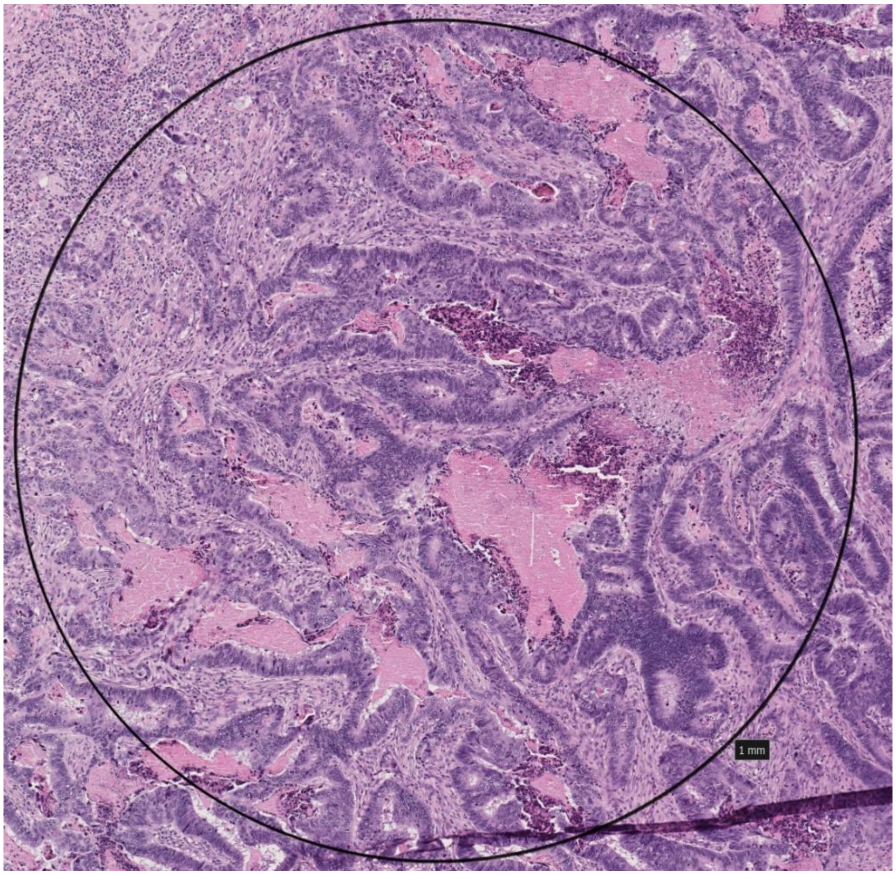

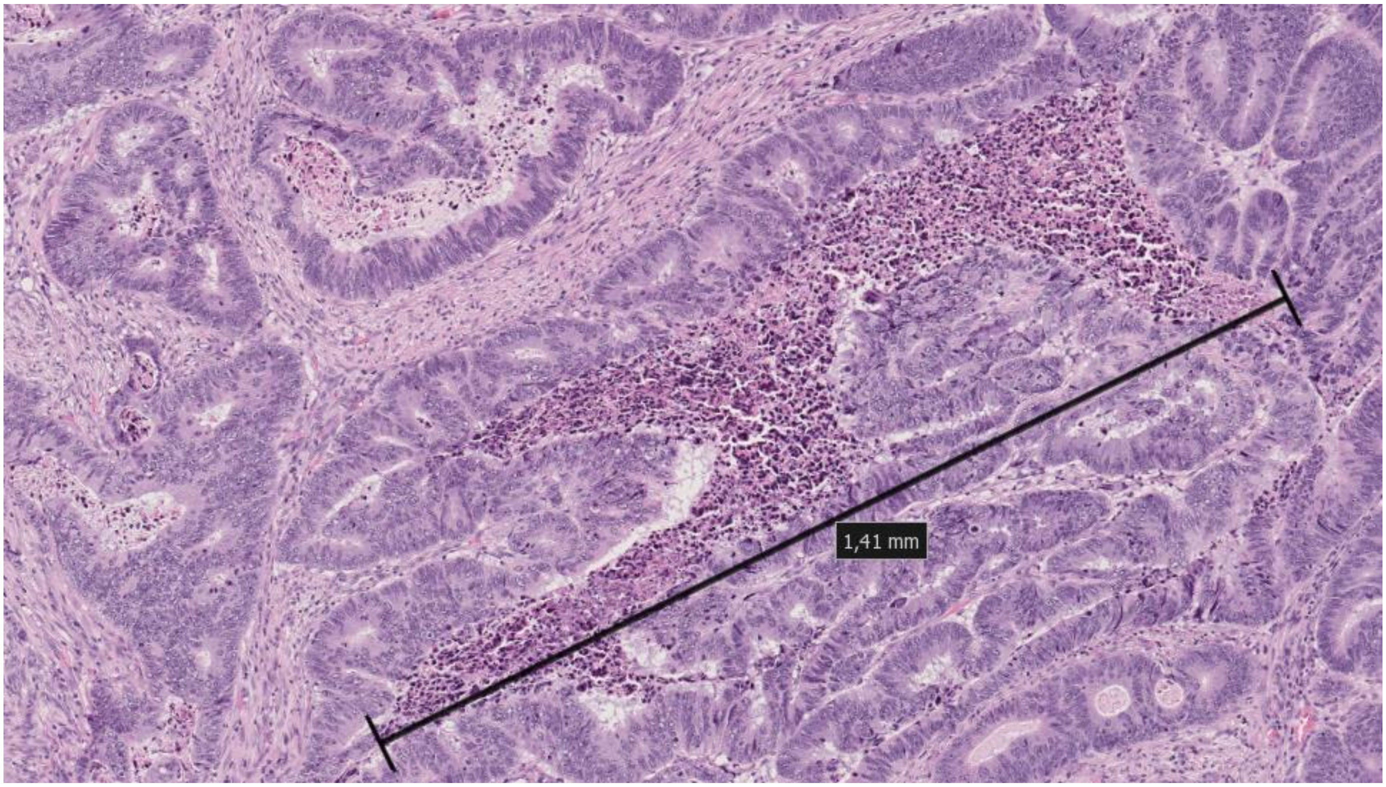

Example 2

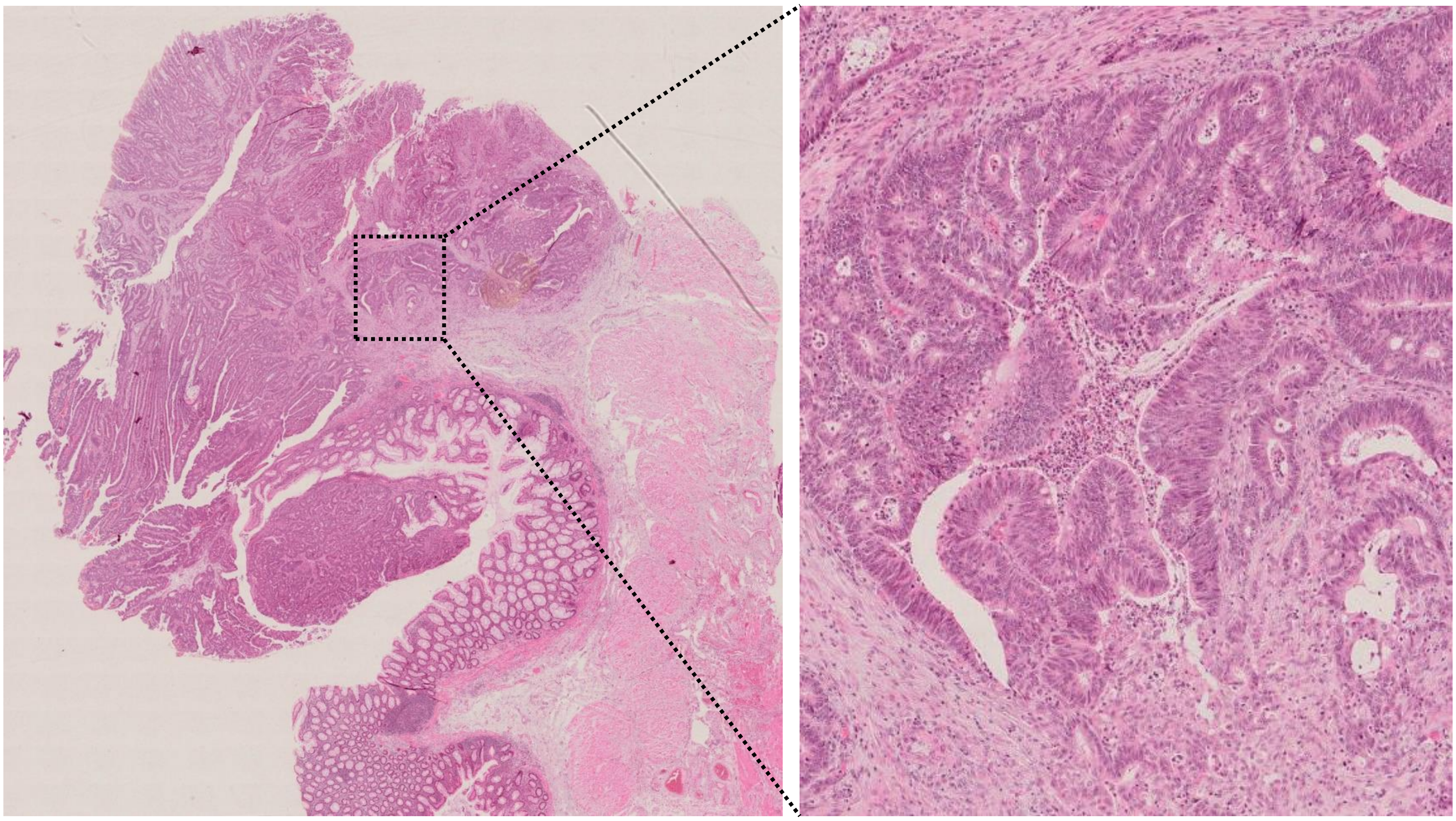

Tumor necrosis evaluation example 2

- Average percentage method: 2% (low)
- Hotspot method: 3% (low)
- Linear method: 433 μm (low)

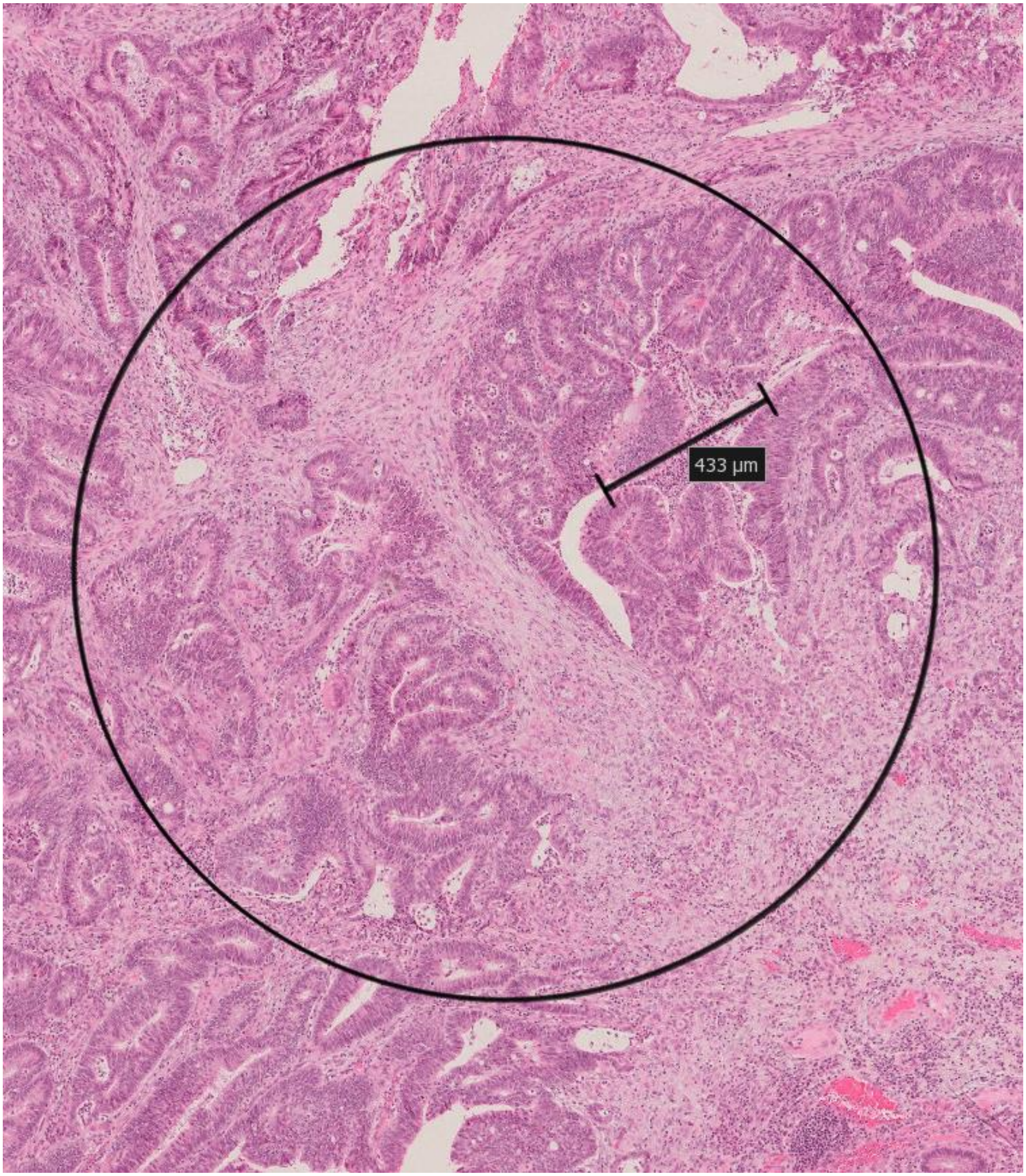

Example 3

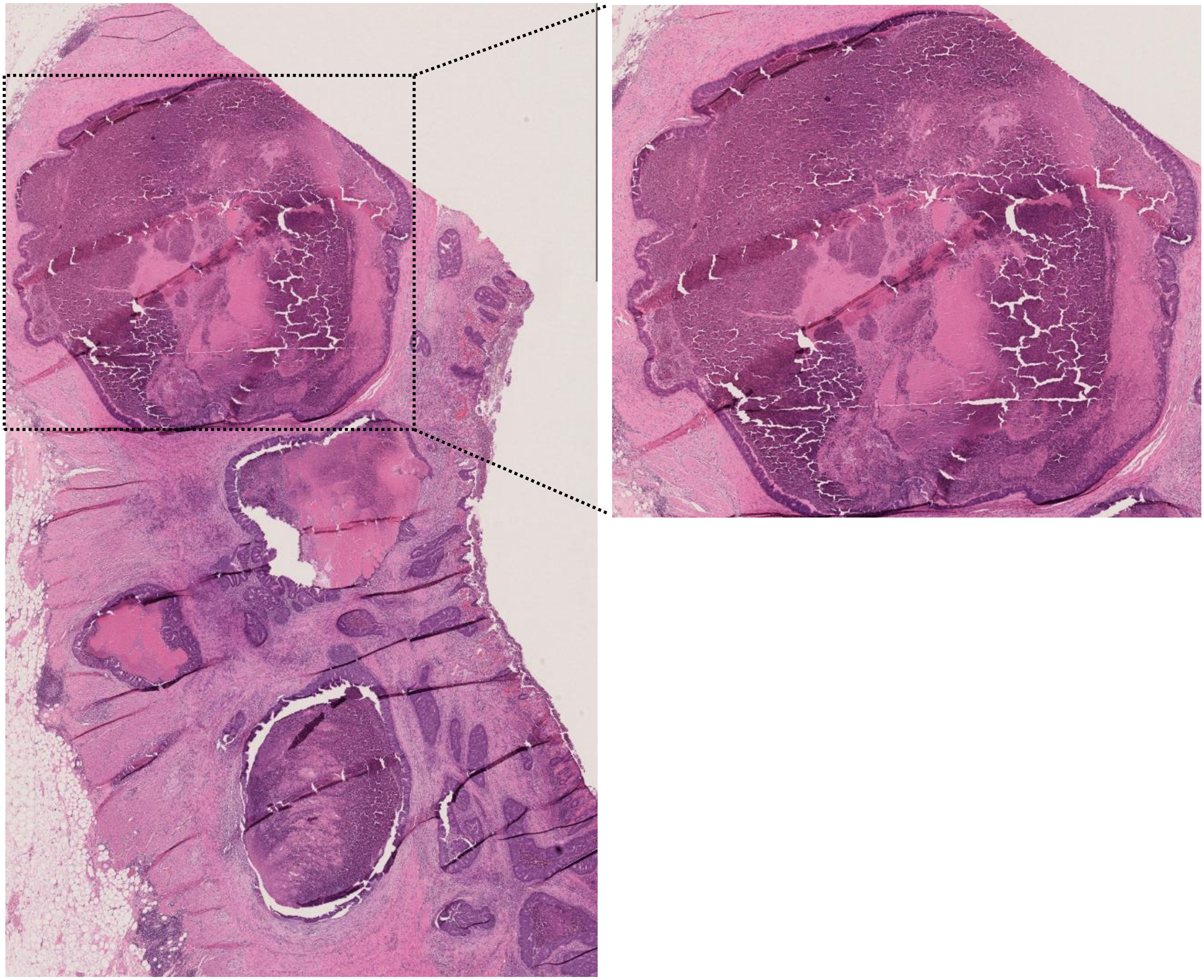

Tumor necrosis evaluation example 3

- Average percentage method: 80% (high)
- Hotspot method: 100% (high)
- Linear method: 5200 µm (high)

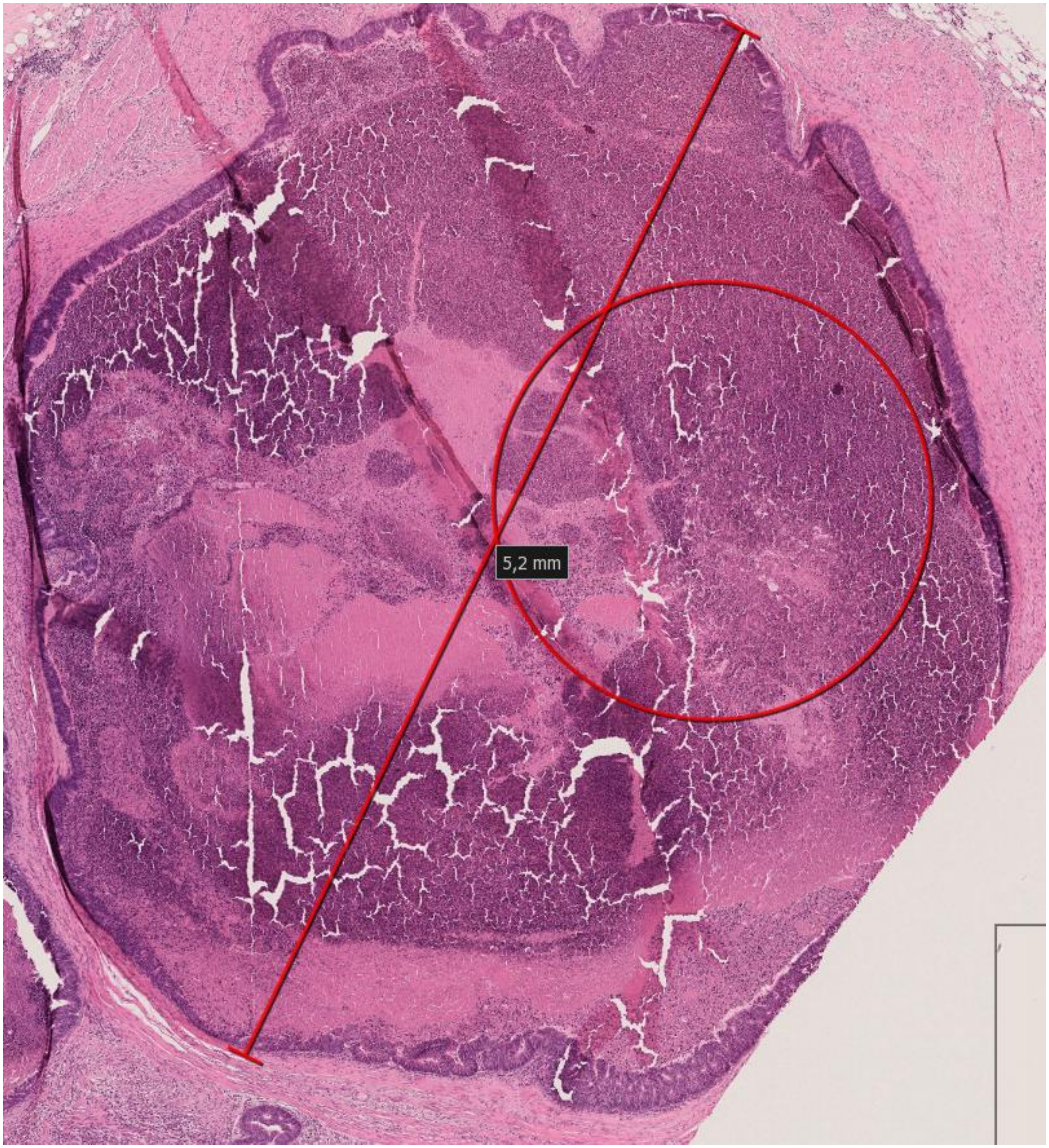

Example 4

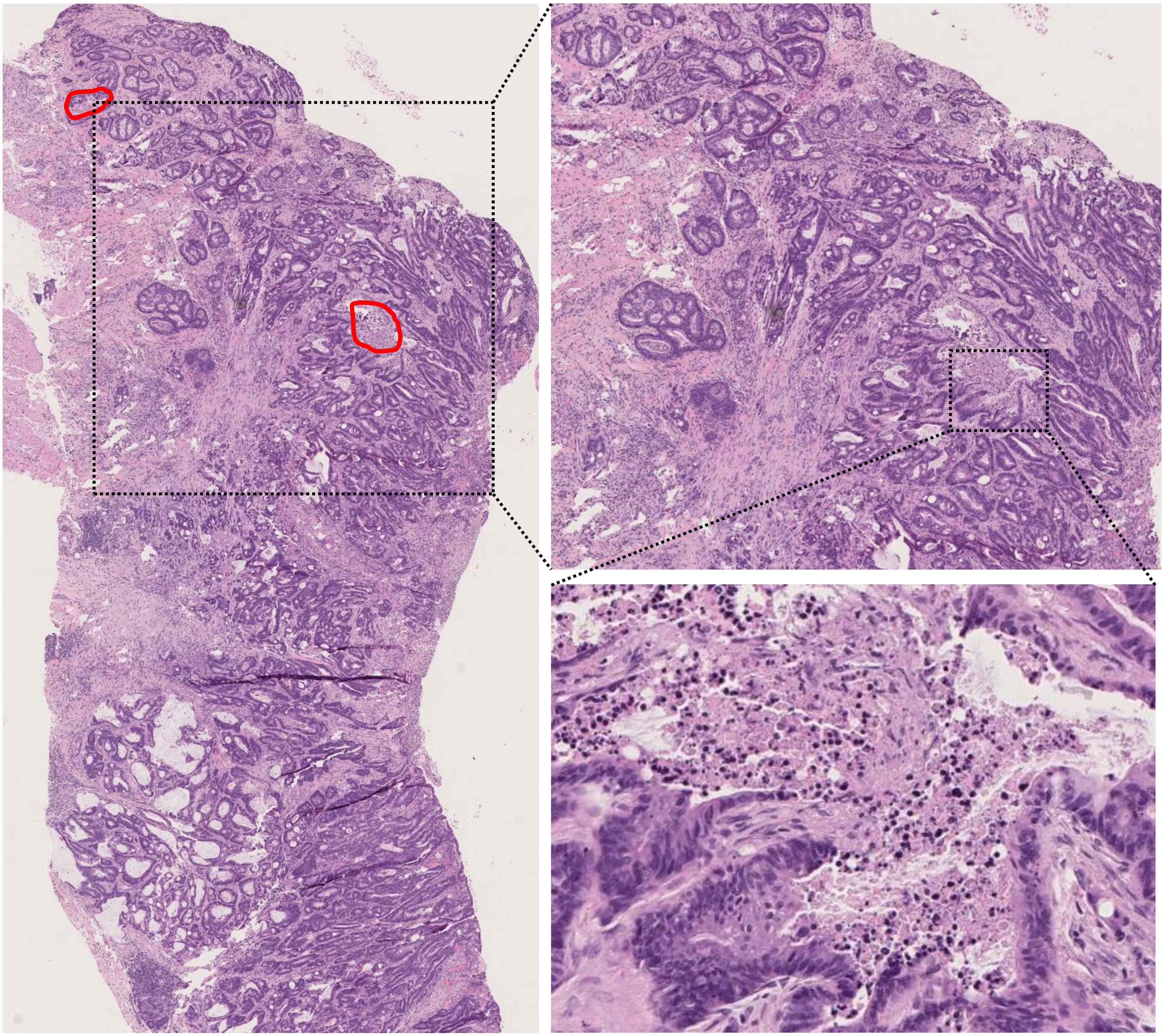

Tumor necrosis evaluation example 4

- Average percentage method: 2% (low)
- Hotspot method: 4% (intermediate)
- Linear method: 674 μm (intermediate)

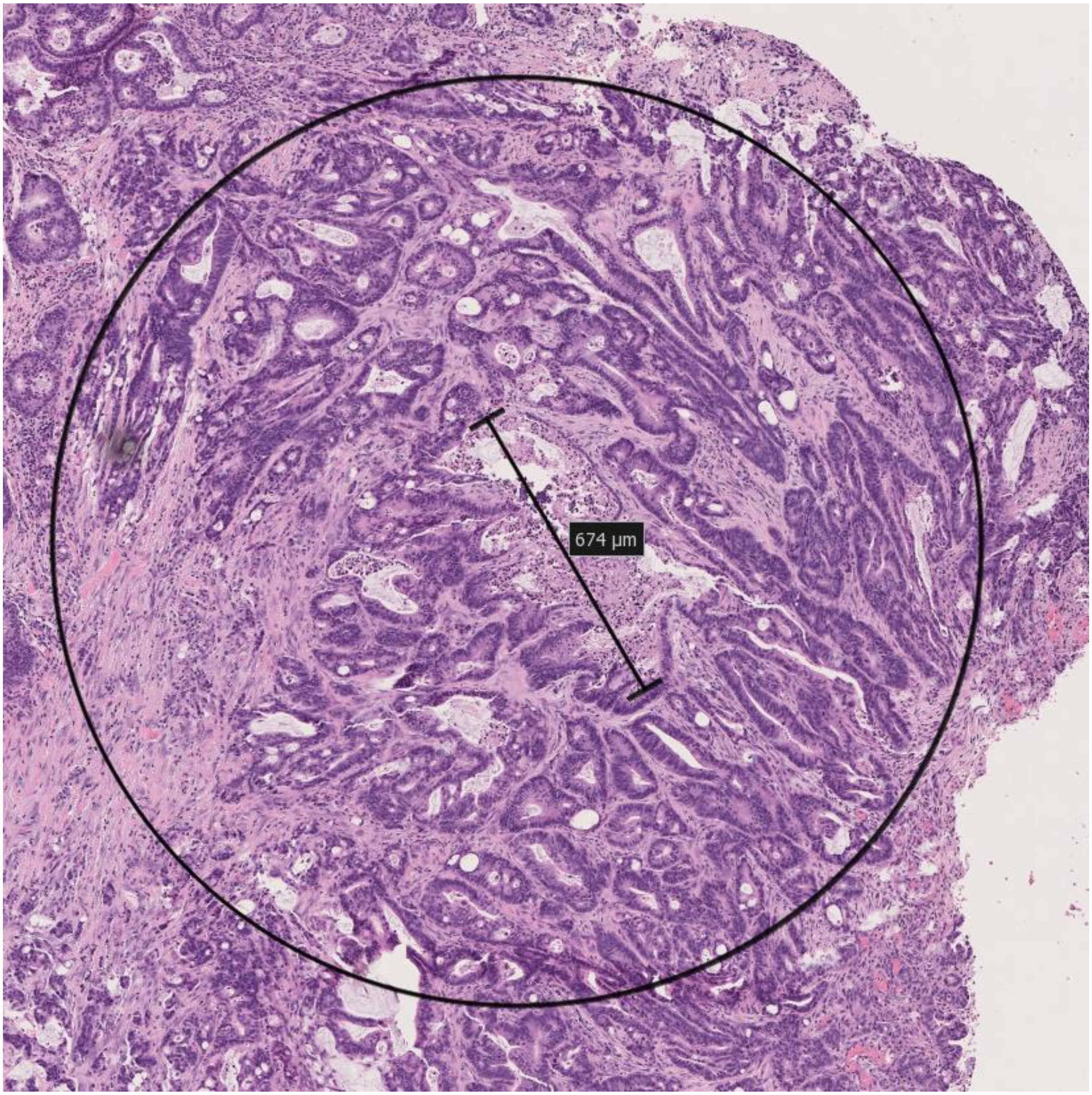

Supplement: SUPPLEMENTARY MATERIAL [file pas-48-1284-s001.pdf]
