# Supplementary material for: Establishing Criteria for Tumor Necrosis as Prognostic Indicator in Colorectal Cancer
Source: Am J Surg Pathol. 2024 Jul 15;48(10):1284–92. doi: 10.1097/PAS.0000000000002286 (PMC11404753; doi:10.1097/PAS.0000000000002286)
Supplement: SUPPLEMENTARY MATERIAL [file pas-48-1284-s002.pdf]

Kastinen M, et al. Establishing criteria for tumor necrosis as prognostic indicator in colorectal cancer. Supplementary table 1.

Table S1. Patient and tumor characteristic according to tumor necrosis hotspot method categories in Cohort 1 and Cohort 2

	Cohort 1 (N=1,100)				Cohort 2 (N=776)			
	Total N(%)	Hotspot method, N (%)			Total N(%)	Hotspot method, N (%)		
**Variable**		<4%	4-79.9%	≥80%		<4%	4-79.9%	≥80%
**Sex**								
Male	557 (51%)	84 (15%)	434 (78%)	39 (7.0%)	412 (53%)	63 (15%)	293 (71%)	56 (14%)
Female	543 (49%)	99 (18%)	405 (75%)	39 (7.2%)	364 (47%)	70 (19%)	238 (65%)	56 (15%)
**P value**		0.358				0.211		
**Age**								
<65	290 (26%)	40 (14%)	230 (79%)	20 (6.9%)	233 (30%)	33 (14%)	168 (72%)	32 (14%)
65-75	381 (35%)	61 (16%)	293 (77%)	27 (7.1%)	285 (37%)	50 (18%)	196 (69%)	39 (14%)
>75	429 (39%)	82 (19%)	316 (74%)	31 (7.2%)	258 (33%)	50 (19%)	167 (65%)	41 (16%)
**P value**		0.425				0.472		
**Tumor location**								
Proximal colon	536 (49%)	112 (21%)	387 (72%)	37 (6.9%)	323 (42%)	68 (21%)	213 (66%)	42 (13%)
Distal colon	404 (37%)	43 (11%)	326 (81%)	35 (8.7%)	205 (26%)	23 (11%)	141 (69%)	41 (20%)
Rectum	160 (15%)	28 (18%)	126 (79%)	6 (3.8%)	248 (32%)	42 (17%)	177 (71%)	29 (12%)
**P value**		<0.001				0.008		
**Stage**								
I	184 (17%)	53 (29%)	129 (70%)	2 (1.1%)	187 (24%)	55 (29%)	129 (69%)	3 (1.6%)
II	408 (37%)	59 (14%)	314 (77%)	35 (8.6%)	253 (33%)	42 (17%)	170 (67%)	41 (16%)
III	355 (32%)	51 (14%)	281 (79%)	23 (6.5%)	251 (32%)	30 (12%)	178 (71%)	43 (17%)
IV	153 (14%)	20 (13%)	115 (75%)	18 (12%)	85 (11%)	6 (7.1%)	54 (64%)	25 (29%)
**P value**		<0.001				<0.001		
**Histological subtype**								
Adenocarcinoma	995 (90%)	137 (14%)	785 (79%)	73 (7.3%)	700 (90%)	100 (14%)	489 (70%)	111 (16%)
Mucinous carcinoma	77 (7.0%)	34 (44%)	40 (52%)	3 (3.9%)	61 (7.9%)	26 (43%)	34 (56%)	1 (1.6%)
Signet ring cell carcinoma	28 (2.5%)	12 (43%)	14 (50%)	2 (7.1)	15 (1.9%)	7 (47%)	8 (53%)	0 (0%)
**P value**		<0.001				<0.001		
**Neuroendocrine differentiation***								
0%	-	-	-	-	560 (72%)	90 (16%)	384 (69%)	86 (15%)
1-9%	-	-	-	-	157 (20%)	27 (17%)	112 (71%)	18 (11%)
≥10%	-	-	-	-	43 (5.5%)	10 (23%)	26 (60%)	7 (16%)
Missing data					16 (2.1%)			
**P value**						0.524		
**WHO grade**								
Low-grade	903 (82%)	129 (14%)	718 (80%)	56 (6.2%)	665 (86%)	106 (16%)	475 (71%)	84 (13%)
High-grade	197 (18%)	54 (27%)	121 (61%)	22 (11%)	111 (14%)	27 (24%)	56 (50%)	28 (25%)
**P value**		<0.001				<0.001		
**Lymphovascular invasion**								
No	858 (78%)	146 (17%)	646 (75%)	66 (7.7%)	429 (55%)	91 (21%)	293 (68%)	45 (10%)
Yes	242 (22%)	37 (15%)	193 (80%)	12 (5.0%)	347 (45%)	42 (12%)	238 (69%)	67 (19%)
**P value**		0.244				<0.001		
**Mismatch repair status**								
MMR proficient	931 (85%)	127 (14%)	742 (80%)	62 (6.7%)	652 (84%)	82 (13%)	472 (72%)	98 (15%)
MMR deficient	169 (15%)	56 (33%)	97 (57%)	16 (9.5%)	124 (16%)	51 (41%)	59 (48%)	14 (11%)
**P value**		<0.001				<0.001		
**BRAF status***								
Wild-type	916 (83%)	128 (14%)	727 (79%)	61 (6.7%)	662 (86%)	91 (14%)	473 (71%)	98 (15%)
Mutant	182 (17%)	54 (30%)	111 (61%)	17 (9.3%)	107 (14%)	40 (37%)	53 (50%)	14 (13%)
Missing cases	2 (0.2%)				7 (0.9%)			
**P value**		<0.001				<0.001		

Abbreviations: MMR, Mismatch repair. *Neuroendocrine differentiation was determined for Cohort 2 using synaptophysin and chromogranin A immunohistochemistry
