# Supplementary material for: Establishing Criteria for Tumor Necrosis as Prognostic Indicator in Colorectal Cancer
Source: Am J Surg Pathol. 2024 Jul 15;48(10):1284–92. doi: 10.1097/PAS.0000000000002286 (PMC11404753; doi:10.1097/PAS.0000000000002286)
Supplement: SUPPLEMENTARY MATERIAL [file pas-48-1284-s004.pdf]

Kastinen M, et al. Establishing criteria for tumor necrosis as prognostic indicator in colorectal cancer. Supplementary figure 1.

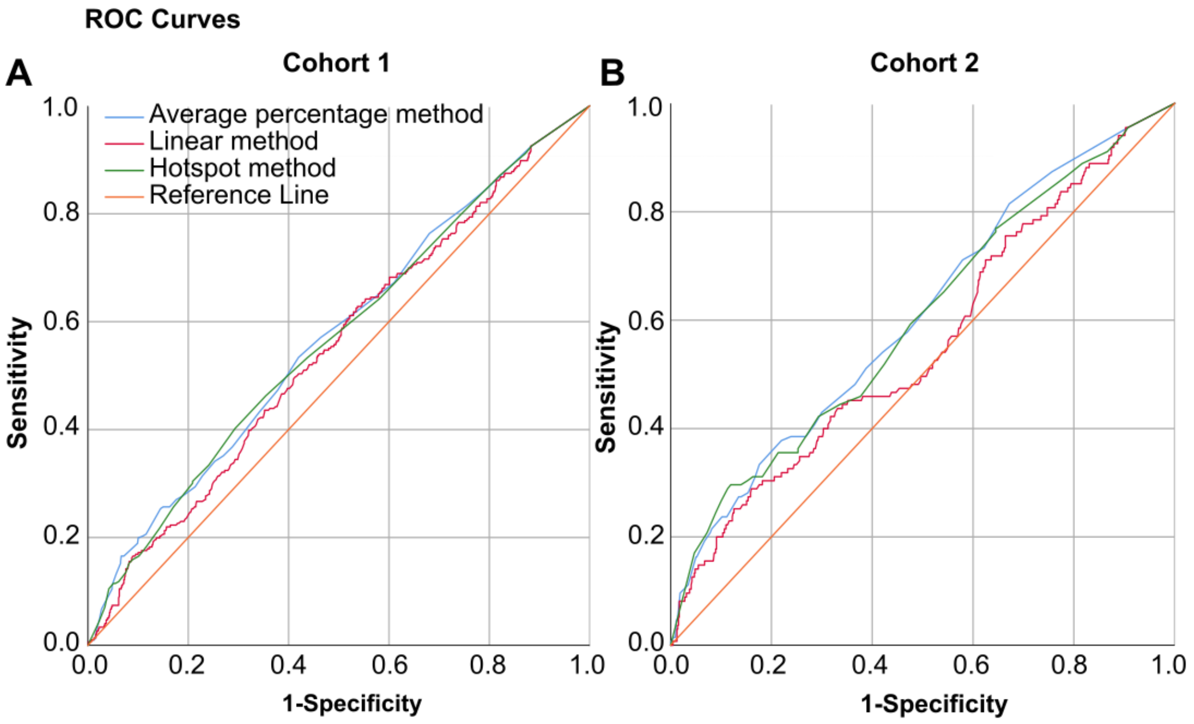

	Cohort 1 Area	CI 95%	Cohort 2 Area	CI 95%
Average percentage method	0.571	0.53-0.63	0.608	0.56-0.66
Linear method	0.550	0.51-0.59	0.558	0.50-0.61
Hotspot method	0.567	0.53-0.61	0.599	0.54-0.65

Figure S1. Receiver operating characteristics (ROC) curves for tumor necrosis evaluation methods in Cohort 1 and 2. A. ROC curve in Cohort 1. **B**. ROC curve in Cohort 2. Abbreviations: CI confidence interval
