# Supplementary material for: Establishing Criteria for Tumor Necrosis as Prognostic Indicator in Colorectal Cancer
Source: Am J Surg Pathol. 2024 Jul 15;48(10):1284–92. doi: 10.1097/PAS.0000000000002286 (PMC11404753; doi:10.1097/PAS.0000000000002286)
Supplement: SUPPLEMENTARY MATERIAL [file pas-48-1284-s005.pdf]

Kastinen M, et al. Establishing criteria for tumor necrosis as prognostic indicator in colorectal cancer. Supplementary table 3

Table S3. Multivariable Cox proportional hazards regression models for average necrosis percentage method.

	Multivariable Hazard ratio (95% CI)	
Variable	Cohort 1	Cohort 2
Average necrosis percentage		
<3%	1 (referent)	1 (referent)
3-39.9%	1.47 (1.07-2.00)	1.11 (0.71-1.74)
≥40%	3.03 (1.93-4.78)	2.97 (1.63-5.40)
Age		
<65	1 (referent)	1 (referent)
65-75	1.23 (0.91-1.65)	1.68 (1.09-2.59)
>75	1.96 (1.46-2.64)	2.49 (1.60-3.89)
Sex		
Male	1 (referent)	1 (referent)
Female	0.89 (0.70-1.13)	0.96 (0.68-1.35)
Year of operation		
2000-2005	1 (referent)	-
2006-2010	0.61 (0.46-0.81)	1 (referent)
2011-2015	0.50 (0.37-0.66)	1.09 (0.72-1.64)
2016-2020	-	0.63 (0.41-0.99)
Tumor location		
Proximal colon	1 (referent)	1 (referent)
Distal colon	0.87 (0.67-1.14)	1.21 (0.79-1.86)
Rectum	0.83 (0.58-1.20)	0.96 (0.62-1.49)
AJCC disease stage		
I-II	1 (referent)	1 (referent)
III	3.02 (2.19-4.15)	2.49 (1.48-4.18)
IV	17.44 (12.33-24.68)	18.31 (10.60-31.63)
Tumor grade		
Low-grade	1 (referent)	1 (referent)
High-grade	2.00 (1.47-2.71)	1.34 (0.86-2.08)
Tumor budding		
Grade 1 (0-4)	1 (referent)	1 (referent)
Grade 2 (5-9)	1.44 (1.06-1.96)	1.76 (1.12-2.75)
Grade 3 (>10)	1.49 (1.09-2.03)	2.27 (1.49-3.44)
Lymphovascular invasion		
No	1 (referent)	1 (referent)
Yes	1.77 (1.37-2.28)	1.77 (1.12-2.08)
Mismatch repair status		
MMR proficient	1 (referent)	1 (referent)
MMR deficient	0.59 (0.36-0.98)	0.53 (0.25-1.14)
BRAF mutation		
Wild-type	1 (referent)	1 (referent)
Mutant	1.35 (0.88-2.07)	1.79 (0.97-3.28)

Abbreviations: MMR, mismatch repair; CI, confidence interval.
