# Supplementary material for: Establishing Criteria for Tumor Necrosis as Prognostic Indicator in Colorectal Cancer
Source: Am J Surg Pathol. 2024 Jul 15;48(10):1284–92. doi: 10.1097/PAS.0000000000002286 (PMC11404753; doi:10.1097/PAS.0000000000002286)
Supplement: SUPPLEMENTARY MATERIAL [file pas-48-1284-s006.pdf]

Kastinen M, et al. Establishing criteria for tumor necrosis as prognostic indicator in colorectal cancer. Supplementary table 4

Table S4. Multivariable Cox proportional hazards regression models for tumor necrosis hotspot method.

	Multivariable Hazard ratio (95% CI)	
Variable	Cohort 1	Cohort 2
Hotspot percentage		
<4%	1 (referent)	1 (referent)
4-79.9%	1.39 (0.97-2.00)	0.84 (0.47-1.51)
≥80%	2.76 (1.72-4.24)	1.82 (0.96-3.44)
Age		
<65	1 (referent)	1 (referent)
65-75	1.21 (0.90-1.64)	1.83 (1.18-2.82)
>75	1.93 (1.44-2.59)	2.73 (1.75-4.26)
Sex		
Male	1 (referent)	1 (referent)
Female	0.87 (0.69-1.11)	1.03 (0.73-1.46)
Year of operation		
2000-2005	1 (referent)	-
2006-2010	0.60 (0.45-0.80)	1 (referent)
2011-2015	0.49 (0.36-0.65)	1.06 (0.70-1.60)
2016-2020	-	0.59 (0.38-0.93)
Tumor location		
Proximal colon	1 (referent)	1 (referent)
Distal colon	0.89 (0.68-1.16)	1.25 (0.81-1.91)
Rectum	0.83 (0.58-1.20)	0.98 (0.63-1.53)
AJCC disease stage		
I-II	1 (referent)	1 (referent)
III	2.98 (2.16-4.10)	2.41 (1.44-4.06)
IV	17.04 (12.06-24.08)	17.77 (10.30-30.66)
Tumor grade		
Low-grade	1 (referent)	1 (referent)
High-grade	1.97 (1.45-2.68)	1.17 (0.75-1.83)
Tumor budding		
Grade 1 (0-4)	1 (referent)	1 (referent)
Grade 2 (5-9)	1.45 (1.07-1.97)	1.77 (1.13-2.77)
Grade 3 (>10)	1.48 (1.08-2.02)	2.16 (1.42-3.29)
Lymphovascular invasion		
No	1 (referent)	1 (referent)
Yes	1.77 (1.37-2.28)	1.94 (1.22-3.08)
Mismatch repair status		
MMR proficient	1 (referent)	1 (referent)
MMR deficient	0.59 (0.35-0.98)	0.56 (0.26-1.21)
BRAF mutation		
Wild-type	1 (referent)	1 (referent)
Mutant	1.32 (0.86-2.02)	1.89 (1.04-3.43)

Abbreviations: MMR, mismatch repair; CI, confidence interval.
