# Supplementary material for: Establishing Criteria for Tumor Necrosis as Prognostic Indicator in Colorectal Cancer
Source: Am J Surg Pathol. 2024 Jul 15;48(10):1284–92. doi: 10.1097/PAS.0000000000002286 (PMC11404753; doi:10.1097/PAS.0000000000002286)
Supplement: SUPPLEMENTARY MATERIAL [file pas-48-1284-s007.pdf]

Kastinen M, et al. Establishing criteria for tumor necrosis as prognostic indicator in colorectal cancer. Supplementary table 5

Table S5. Multivariable Cox proportional hazards regression models for tumor necrosis linear method.

	Multivariable Hazard ratio (95% CI)	
Variable	Cohort 1	Cohort 2
Linear method		
≤500 μm	1 (referent)	1 (referent)
501-3500 μm	1.36 (1.02-1.80)	1.24 (0.72-2.13)
>3500μm	2.06 (1.34-3.17)	1.96 (1.06-3.62)
Age		
<65	1 (referent)	1 (referent)
65-75	1.20 (0.89-1.62)	1.74 (1.13-2.68)
>75	1.86 (1.39-2.49)	2.60 (1.67-4.06)
Sex		
Male	1 (referent)	1 (referent)
Female	0.88 (0.69-1.12)	1.00 (0.71-1.41)
Year of operation		
2000-2005	1 (referent)	-
2006-2010	0.61 (0.46-0.80)	1 (referent)
2011-2015	0.47 (0.35-0.63)	1.04 (0.69-1.58)
2016-2020	-	0.56 (0.36-0.87)
Tumor location		
Proximal colon	1 (referent)	1 (referent)
Distal colon	0.86 (0.66-1.13)	1.25 (0.82-1.93)
Rectum	0.83 (0.58-1.20)	0.98 (0.63-1.53)
AJCC disease stage		
I-II	1 (referent)	1 (referent)
III	2.98 (2.16-4.10)	2.34 (1.42-4.01)
IV	17.18 (12.16-24.26)	17.45 (10.10-30.12)
Tumor grade		
Low-grade	1 (referent)	1 (referent)
High-grade	1.95 (1.44-2.65)	1.28 (0.83-1.98)
Tumor budding		
Grade 1 (0-4)	1 (referent)	1 (referent)
Grade 2 (5-9)	1.43 (1.05-1.93)	1.75 (1.11-2.74)
Grade 3 (>10)	1.51 (1.10-2.06)	2.15 (1.41-3.27)
Lymphovascular invasion		
No	1 (referent)	1 (referent)
Yes	1.77 (1.38-2.28)	1.90 (1.20-3.01)
Mismatch repair status		
MMR proficient	1 (referent)	1 (referent)
MMR deficient	0.59 (0.35-0.97)	0.59 (0.28-1.25)
BRAF mutation		
Wild-type	1 (referent)	1 (referent)
Mutant	1.37 (0.90-2.11)	1.90 (1.04-3.49)

Abbreviations: MMR, mismatch repair; CI, confidence interval.
