# Supplementary material for: Establishing Criteria for Tumor Necrosis as Prognostic Indicator in Colorectal Cancer
Source: Am J Surg Pathol. 2024 Jul 15;48(10):1284–92. doi: 10.1097/PAS.0000000000002286 (PMC11404753; doi:10.1097/PAS.0000000000002286)
Supplement: SUPPLEMENTARY MATERIAL [file pas-48-1284-s008.pdf]

Kastinen M, et al. Establishing criteria for tumor necrosis as prognostic indicator in colorectal cancer. Supplementary figure 2.

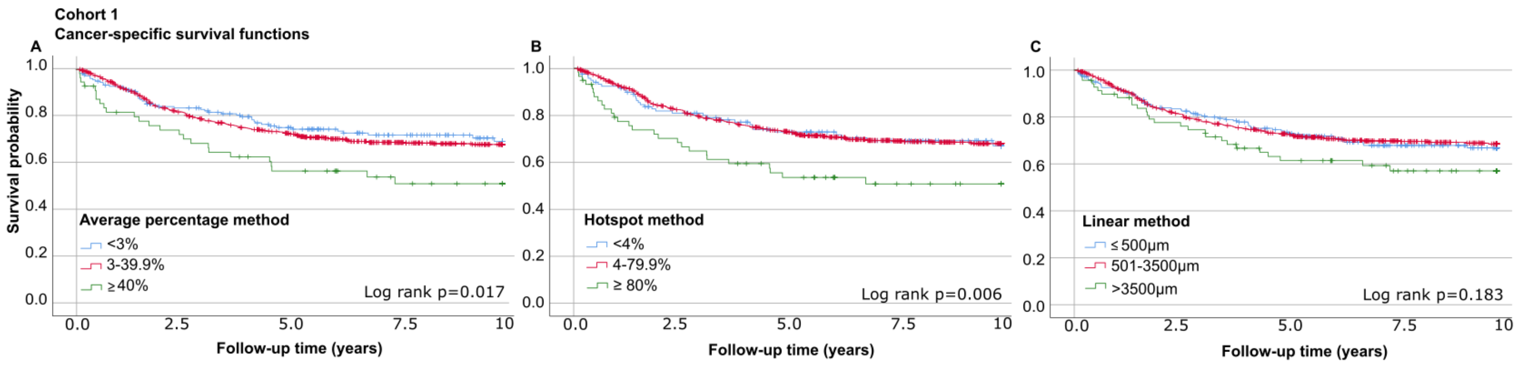

Average percentage method	Years, number at risk				
	0	2.5	5	7.5	10
<3%	182	140	108	75	47
3-39.9%	662	499	408	277	184
≥40%	56	38	28	18	14

Hotspot method	Years, number at risk				
	0	2.5	5	7.5	10
<4%	121	91	70	51	31
4-79.9%	719	547	446	302	202
≥80%	60	39	28	17	12

Linear method	Years, number at risk				
	0	2.5	5	7.5	10
≤500 μm	220	169	134	86	53
501-3500 μm	611	457	375	259	176
>3500 μm	69	51	35	25	16

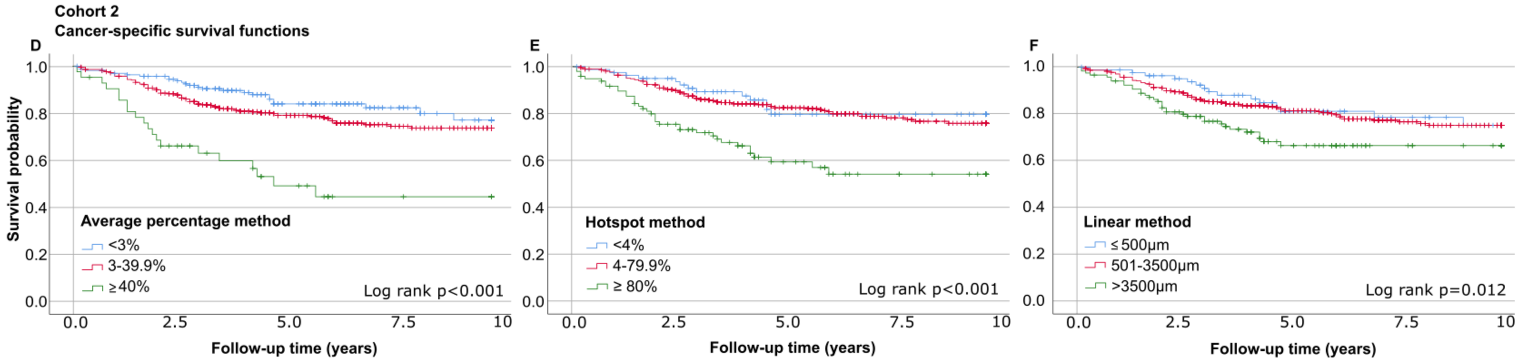

Average percentage method	Years, number at risk				
	0	2.5	5	7.5	10
<3%	169	148	81	43	24
3-39.9%	436	359	205	111	71
≥40%	43	26	13	5	4

Hotspot method	Years, number at risk				
	0	2.5	5	7.5	10
<4%	81	68	35	25	16
4-79.9%	470	399	236	121	75
≥80%	97	66	28	13	8

Linear method	Years, number at risk				
	0	2.5	5	7.5	10
≤500 μm	84	71	40	28	18
501-3500 μm	446	375	221	117	72
>3500 μm	118	87	38	14	9

Figure S2. Kaplan-Meier curves for the three necrosis estimation methods in mismatch repair proficient cases of Cohort 1 and Cohort 2.
