# Supplementary material for: Establishing Criteria for Tumor Necrosis as Prognostic Indicator in Colorectal Cancer
Source: Am J Surg Pathol. 2024 Jul 15;48(10):1284–92. doi: 10.1097/PAS.0000000000002286 (PMC11404753; doi:10.1097/PAS.0000000000002286)
Supplement: SUPPLEMENTARY MATERIAL [file pas-48-1284-s009.pdf]

Kastinen M, et al. Establishing criteria for tumor necrosis as prognostic indicator in colorectal cancer. Supplementary figure 3

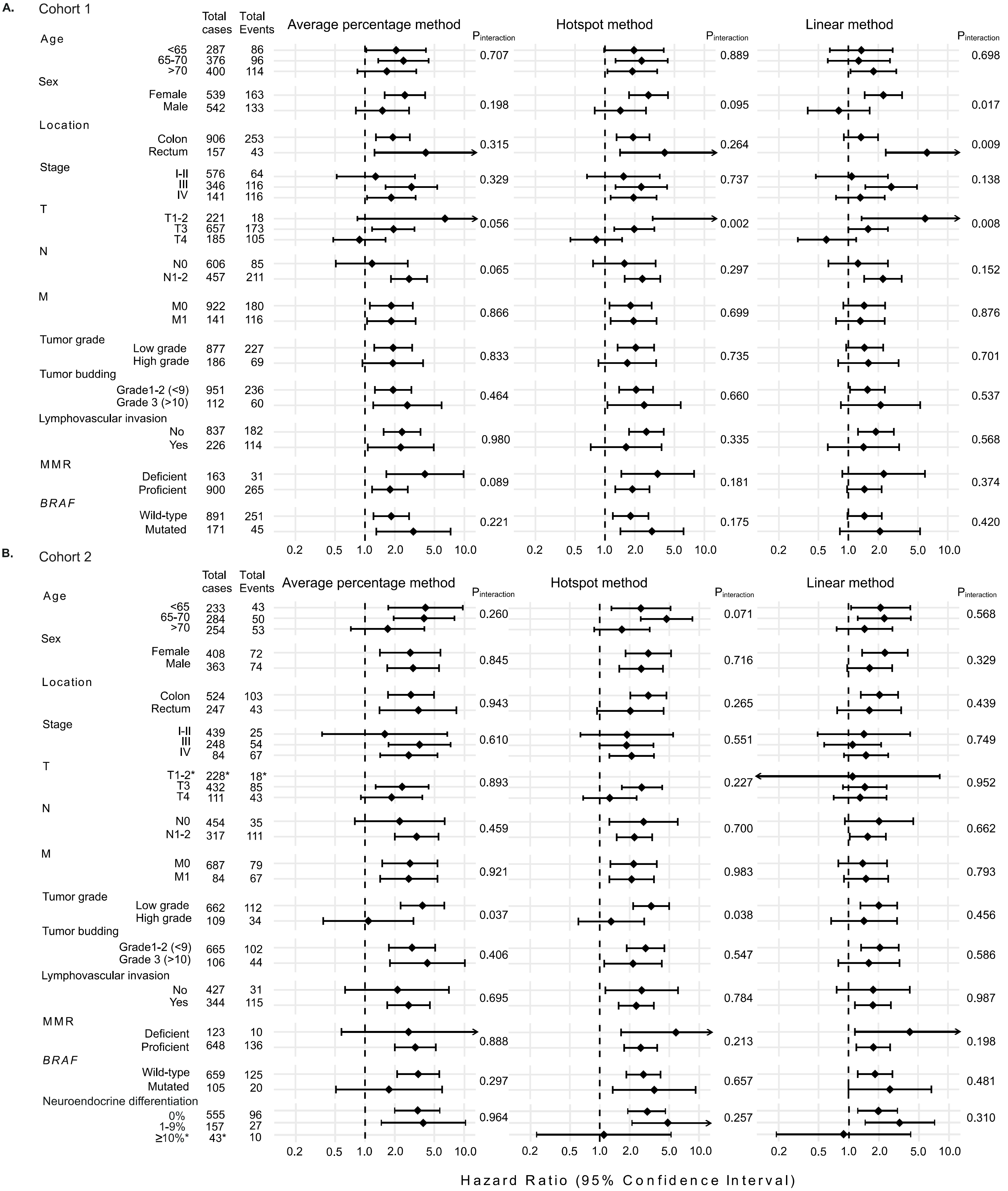

Figure S3. Forest plot subgroup analysis for necrosis evaluation methods in **A.** Cohort 1 and **B.** Cohort 2. Abbreviations: MMR, mismatch repair. Neuroendocrine differentiation was determined for Cohort 2 using synaptophysin and chromogranin A immunohistochemistry.

* Too few cases in the highest tumor necrosis category (average percentage method and hotspot method) in T1-2 tumors, as well as in neuroendocrine differentiation ≥10% (average percentage method), for reliable analysis.
